# *Aspergillus terreus* sectorization: a morphological phenomenon shedding light on amphotericin B resistance mechanism

**DOI:** 10.1128/mbio.03926-24

**Published:** 2025-02-25

**Authors:** David Eisele, Michael Blatzer, Anna Maria Dietl, Ulrike Binder, Christoph Müller, Ferry Hagen, Tongta Sae-Ong, Sascha Schäuble, Gianni Panagiotou, Roya Vahedi-Shahandashti, Cornelia Lass-Flörl

**Affiliations:** 1 Institute of Hygiene and Medical Microbiology, Medical University of Innsbruck 27280 , Innsbruck, Austria; 2 Experimental Neuropathy Unit, Institute Pasteur, Paris, France; 3 Global Health Department, Institute Pasteur, Paris, France; 4 Department of Pharmacy-Center for Drug Research, Ludwig-Maximilians-Universität München, Munich, Germany; 5 Department of Medical Mycology, Westerdijk Fungal Biodiversity Institute (WI-KNAW), Utrecht, the Netherlands; 6 Institute of Biodiversity and Ecosystem Dynamics (IBED), University of Amsterdam, Amsterdam, the Netherlands; 7 Department of Medical Microbiology, University Medical Center Utrecht, Utrecht, the Netherlands; 8 Department of Microbiome Dynamics, Leibniz Institute for Natural Product Research and Infection Biology – Hans Knöll Institute (Leibniz-HKI), Jena, Germany; 9 Faculty of Biological Sciences, Friedrich Schiller University, Jena, Germany; 10 Jena University Hospital, Friedrich Schiller University, Jena, Germany; 11 Cluster of Excellence Balance of the Microverse, Friedrich Schiller University, Jena, Germany; Vallabhbhai Patel Chest Institute, Delhi, India

**Keywords:** *Aspergillus terreus*, P-type ATPase, amphotericin B (AmB) resistance, culture degeneration, sectorization, phenotypic heterogeneity, polyketide synthases (PKS)

## Abstract

Prolonged cultivation of certain filamentous fungi, including *Aspergillus terreus*, on drug-free medium leads to degeneration and morphological heterogeneity, marked by the emergence of fluffy mycelium-type sectors. This phenomenon may indicate alterations in antifungal susceptibility profiles (particularly to amphotericin B (AmB) in *A. terreus*), as well as reductions or losses in conidiation, sexuality, secondary metabolite production, and/or virulence. In the present study, various characteristics of an AmB-resistant wild-type (WT) strain and its AmB-susceptible sectorized derivative (ATSec) were characterized. Compared to WT, ATSec exhibited increased susceptibility to AmB, reduced sporulation, and comparable sterol contents and virulence in *Galleria mellonella*. To elucidate the genes involved in AmB resistance, gene expression levels were compared between WT and ATSec with and without AmB treatment. The expression of P-type ATPase-related genes, which are implicated in membrane composition changes and consequently in AmB resistance, was significantly higher in the WT strain compared to ATSec. Moreover, the up-regulation of genes involved in the biosynthesis of polyketides—a diverse group of secondary metabolites—was higher in WT compared to ATSec, with a significant number of these genes also carrying at least one mutation. The findings of this study indicate that P-type ATPases may significantly be involved in AmB susceptibility and resistance observed in ATSec and WT strains. Additionally, mutations in polyketide synthase genes in ATSec may contribute to the phenotypic alterations associated with the sectorized phenotype.

**IMPORTANCE:**

Prolonged cultivation of certain filamentous fungi, including Aspergillus terreus, on drug-free medium leads to degeneration and morphological heterogeneity, marked by the emergence of fluffy mycelium-type sectors. This phenomenon may indicate alterations in antifungal susceptibility profiles (particularly to amphotericin B (AmB) in A. terreus), as well as reductions or losses in conidiation, sexuality, secondary metabolite production, and/or virulence. In the present study, various characteristics of an AmB-resistant wild-type strain (WT) and its AmB-susceptible sectorized derivative (ATSec) were characterized. Compared to WT, ATSec exhibited increased susceptibility to AmB, reduced sporulation, and comparable sterol contents and virulence in Galleria mellonella. To elucidate the genes involved in AmB resistance, gene expression levels were compared between WT and ATSec with and without AmB treatment. The expression of P-type ATPase-related genes, which are implicated in membrane composition changes and consequently in AmB resistance, was significantly higher in the WT strain compared to ATSec. Moreover, the up-regulation of genes involved in the biosynthesis of polyketides - a diverse group of secondary metabolites - was higher in WT compared to ATSec, with a significant number of these genes also carrying at least one mutation. The findings of this study indicate that P-type ATPases may significantly be involved in AmB susceptibility and resistance observed in ATSec and WT strains. Additionally, mutations in polyketide synthase genes in ATSec may contribute to the phenotypic alterations associated with the sectorized phenotype.

## INTRODUCTION

Filamentous fungi are known for altering their morphology when repeatedly subcultured on artificial media; this is called culture degeneration, phenotypic instability, deterioration, and dual phenomenon (1, 2). Classically, this involves alterations in color; growth form with a fluffy/woolly appearance; reduced sporulation, as well as a reduction or loss of secondary metabolite production; and other irreversible phenotypes (1, 3). So far, most studies were focused on the industrial rather than the medical setting (3, 4) and the few clinical studies investigating sectorization, focused on yeasts rather than molds (5). Sectorization is of relevance to the industry because the resulting reduction in (secondary) metabolite production causes financial losses. Danner et al. (4) describe sectorization affecting citric acid production in *Aspergillus niger* and hydrolytic enzyme production in *Aspergillus oryzae*.

The underlying molecular mechanism of this unique trait remains not fully understood, with a current study discussing genetic, epigenetic, and stress-related mechanisms (4). In an interesting parallel to sectorization, mutations in polyketide synthases (PKS) were discovered to impact pigmentation, sporulation/conidiation, and virulence in different species (6–9). Our previous studies have demonstrated how culture degeneration in *Aspergillus terreus* can impact antifungal susceptibility testing (AFST) profiles and virulence traits (10), prompting the present study. The degenerated culture of *A. terreus*, referred to as sector (ATSec), exhibited greater virulence, and increased susceptibility to amphotericin B (AmB) compared to the non-degenerated part of the culture, wild-type (WT), but remained unaffected by other tested antifungal classes (10). *A. terreus* is clinically important due to its natural reduced susceptibility to AmB, known as intrinsic resistance (11); however, some rare strains of *A. terreus* are susceptible or tolerant to AmB (12).

The precise mechanism of action of AmB is not fully understood, but it is known that AmB forms 1:1 adducts with ergosterol, forming non-aqueous pores in fungal cell membranes that cause membrane permeation and ion leakage, as well as ergosterol depletion/reduction due to 1:1 adducts, both contributing to AmB’s toxicity (11, 13). Pore formation enhances AmB activity, but alone is insufficient to induce cell killing, indicating it serves as a secondary mechanism, with AmB likely employing additional killing mechanisms (14). Intracellularly, AmB induces oxidative stress (11, 13). However, this intracellular effect also requires binding to ergosterol (11, 13). It is postulated that mitochondrial activity is also influenced by AmB, as reactive oxygen species (ROS) generation is a byproduct of the mitochondrial respiratory chain (11, 15). The oxidative burst can damage cellular components and cause cell death (11, 13). To date, a variety of resistance mechanisms to AmB have been identified in different fungal species (11, 13). Firstly, alterations in sterol composition reduce AmB binding (11). However, some studies indicate that ergosterol content plays a minor role in intrinsic resistance in species like *A. terreus* and does not directly correlate with intracellular AmB levels (16). Secondly, changes in cell wall components contribute to AmB resistance (17). Thirdly, oxidative stress responses have been implicated in AmB resistance, particularly in *A. terreus*, where increased activities of superoxide dismutase and catalase enhance the organism’s ability to counteract oxidative stress induced by the drug (15, 18). Additionally, disruption of mitochondrial function influences free radical production and ergosterol biosynthesis, further contributing to AmB resistance (19). Moreover, changes in membrane composition can influence AmB resistance (13, 20), and a P-type ATPase in protozoan parasites, namely, *Leishmania*, has been shown to impact both membrane composition and AmB resistance (21). P-type ATPases constitute a large, ancient superfamily of primary active pumps that transport a wide range of substrates, from hydrogen ions (H^+^) to phospholipids, across cell membranes against a concentration gradient, utilizing energy from ATP hydrolysis (22). P-type ATPases also play a role in detoxification (23, 24), and links between mutations in P-type ATPase and changes in membrane composition have been observed (21).

The increased AmB susceptibility observed in ATSec compared to the AmB-resistant WT in our previous study (10) provides the direction for the current investigation, focusing on understanding how culture degeneration could render AmB-resistant strains susceptible. To investigate this question, the spontaneous sterile sector derived from the ATSec was subcultured and characterized alongside the WT in the current study. To determine whether AmB susceptibility in ATSec is influenced by genomic mutations, transcriptional changes, or both, whole-genome sequencing and transcriptional profiling were conducted and compared to the WT strain post-AmB exposure.

## RESULTS

### Distinct morphological features of ATSec compared to WT

**Fig 1:**
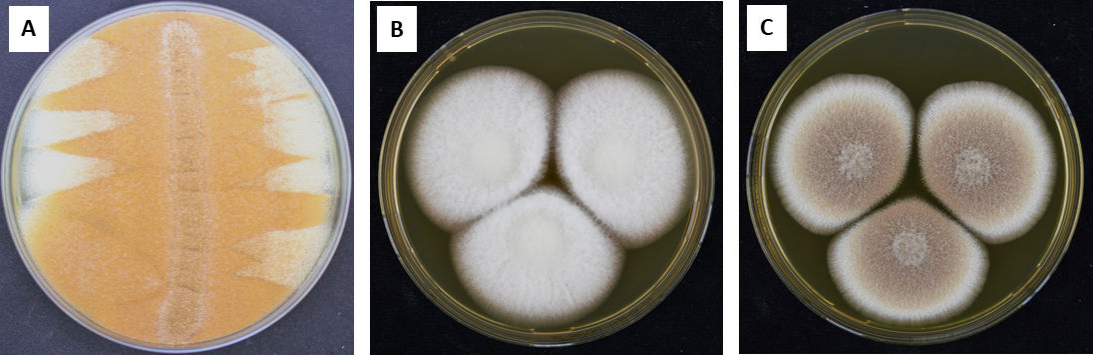
Morphological features of *A. terreus* WT isolate vs ATSec. WT isolate exhibiting culture degeneration (sector formation) after 12 days of growth on SAB medium at 37°C (**A**), sub-cultivations of ATSec (**B**), and WT (**C**), 7-day-old cultures on malt extract agar at 37°C.

**Fig 2:**
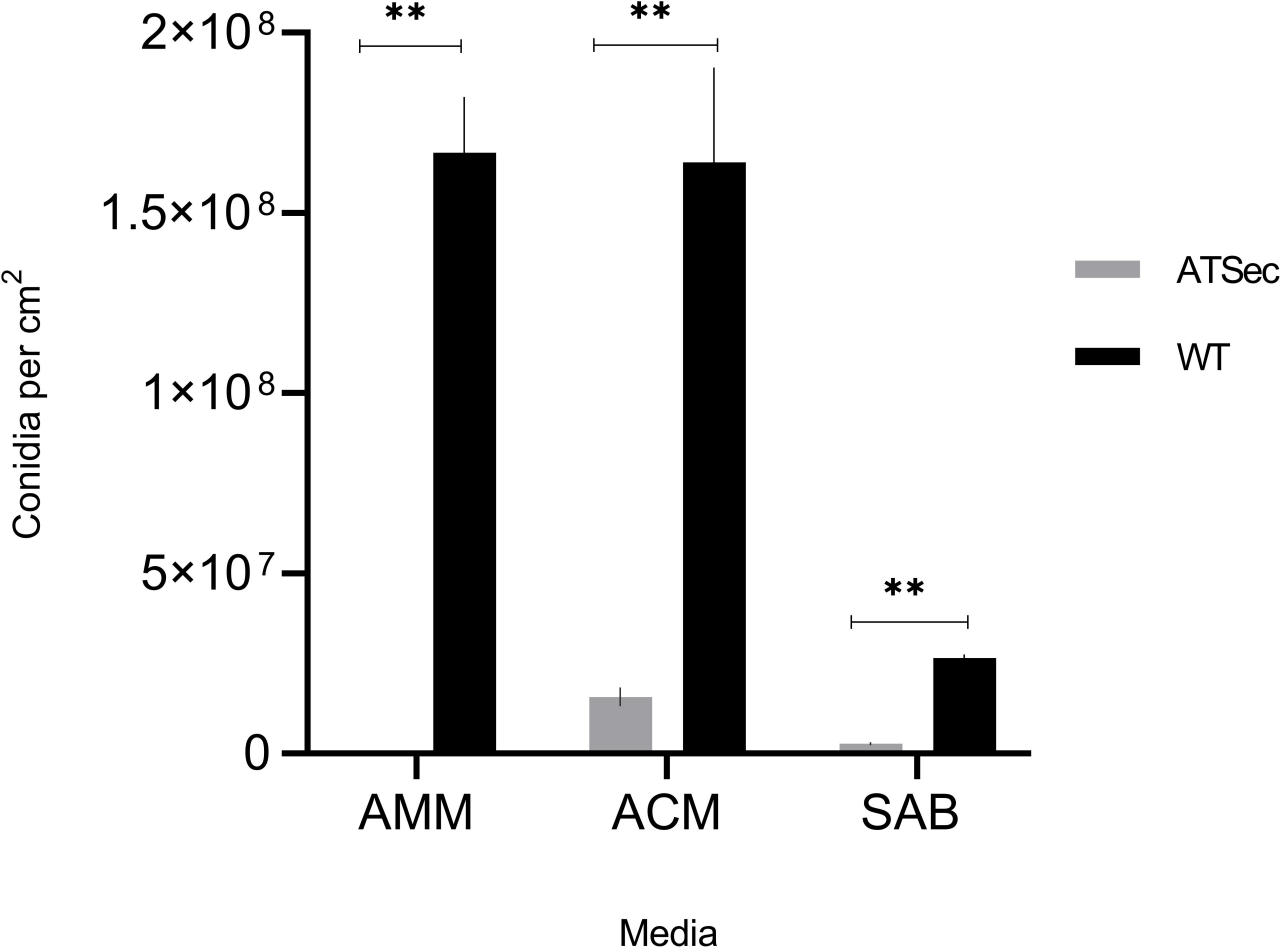
Comparison of sporulation of *A. terreus* WT vs ATSec. WT and ATSec were cultured on AMM, ACM, and SAB medium for 7 days at 37°C. Each value represents mean ± SD of three biological replicates. Statistical analyses were performed using a paired *t*-test. (** *P* < 0.01).

*A. terreus* WT formed multiple sectors simultaneously at the colony margin (Fig. 1A). The isolated ATSec compared to WT exhibited the following characteristics: (i) an irreversible morphological change, with a cotton-like appearance and reduced pigmentation (Fig. 1B) compared to WT (Fig. 1C); (ii) a different AmB MIC range according to EUCAST, WT (2–4 mg/L) and ATSec (1–2 mg/L); and (iii) reduced sporulation (Fig. 2) regardless of the medium used, including Sabouraud dextrose agar (SAB), *Aspergillus* complete medium (ACM), and *Aspergillus* minimal medium (AMM).

### No virulence difference in *G. mellonella* model

**Fig 3:**
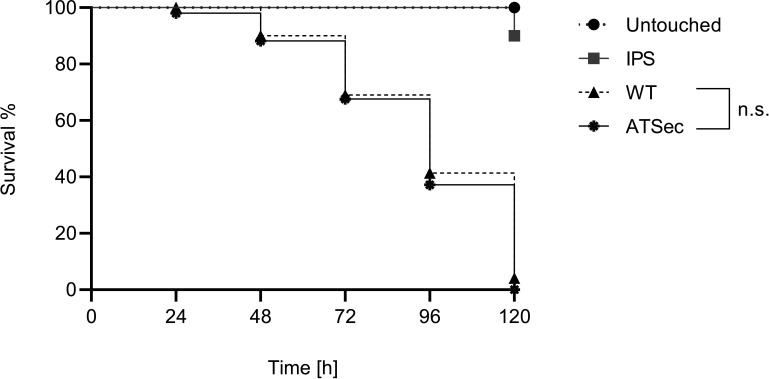
Median survival curves of *A. terreus* WT vs ATSec. Larvae were infected with 2 × 10^6^ spores/larva of WT and ATSec. Control groups included untouched larvae and larvae inoculated with IPS. Survival was monitored every 24 h over a 120-h period. Median survival rates were calculated from two independent experiments, each with three biological replicates (20 larvae per each biological replicates, 120 larvae in total). *P*-values indicating significant differences (*P* < 0.05; Mantel-Cox test) are shown; otherwise, results are marked as not significant (n.s.).

There was no significant difference in the survival curves of *G. mellonella* infected with either WT or the ATSec strain (Fig. 3).

### Sterol content analysis

To investigate potential differences in sterol content, sterol analysis on ATSec and WT was performed using gas chromatography-mass spectrometry (GC-MS). A total of 11 different sterols were detected, and no significant differences in sterol content were observed between WT and ATSec (Table 1).

**TABLE 1:** Sterol composition/content of *A. terreus* WT and ATSec^*a*^

Compound (IUPAC name)	WT		ATSec	
	Relative [%]	Absolute [µg/mg]	Relative [%]	Absolute [µg/mg]
Ergosta-5,8,22-trienol (Lichesterol)	1.4	0.02 (± 0.00)	1.3	0.02 (± 0.01)
Ergosta, 5,7,22-trienol (Ergosterol)	87.5	1.31 (± 0.00)	90.1	1.71 (± 0.66)
Ergosta-5,7,24 (28)-trienol (5-Dedydroepisterol)	1.4	0.02 (± 0.00)	0.7	0.01 (± 0.00)
Ergosta-5,8,22,24 (28)-tetraen-ol	1.9	0.03 (± 0.00)	2.0	0.03 (± 0.01)
Ergosta-5,7-dienol	2.3	0.03 (± 0.00)	0.6	0.01 (± 0.00)
Ergosta-7,22,24 (28)-trienol	0.1	0.00 (± 0.00)	0.2	0.00 (± 0.00)
ergosta-7,24 (28)-dienol (episterol)	2.5	0.04 (± 0.00)	1.3	0.02 (± 0.01)
4,4,14-Trimethylcholesta-8,24 (28)-dienol (Lanosterol)	1.0	0.02 (± 0.00)	1.0	0.02 (± 0.01)
4-Methylergosta-8,24 (28)-dienol (4-Methylfecosterol)	0.4	0.01 (± 0.00)	0.7	0.01 (± 0.00)
4,4,14-Trimethylergosta-8,24 (28)-dienol (Eburicol)	1.4	0.02 (± 0.00)	2.5	0.04 (± 0.01)
4,4-Dimethylergosta-8,24 (28)-dienol	0.6	0.01 (± 0.00)	0.9	0.02 (± 0.01)

^*a*^ Cultures were grown in Sabouraud dextrose broth medium 16 h at 30°C. Sterol composition is given as the relative amount of the respective sterol (%) of all sterols detected. Sterol content is expressed as µg sterol intermediate/mg biomass (dry weight). The results are presented as the average of two technical replicas of two independent experiments. Standard deviation is given in brackets.

### Significant difference in mapping rates between WT and ATSec

**Fig 4:**
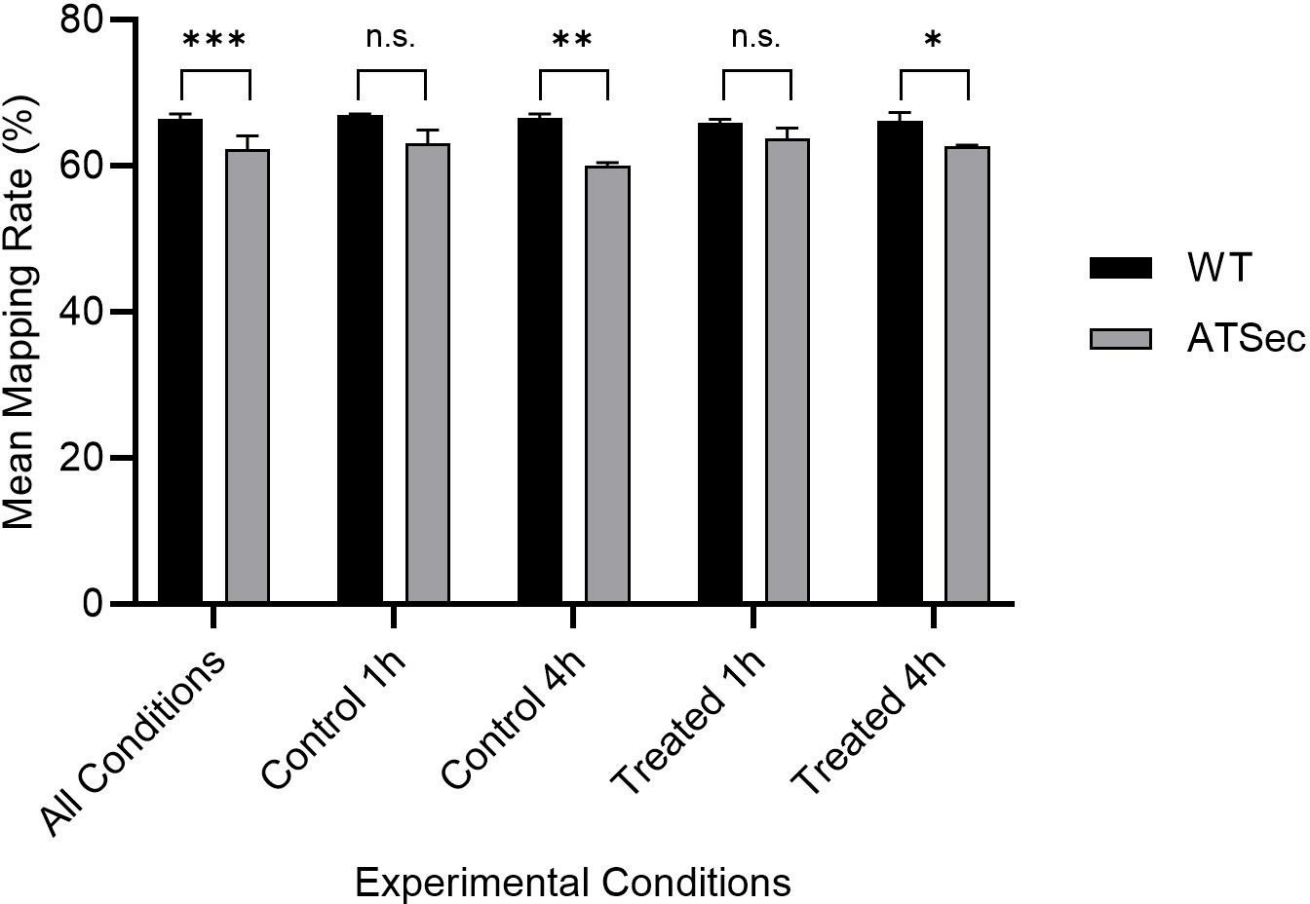
Mean mapping rates. The All Conditions category is the average Salmon mapping rate of a genotype (WT or its sectorized derivative [ATSec]) across all samples, while the control 1 h, control 4 h, treated 1 h, and treated 4 h categories are the average Salmon mapping rate of a genotype (WT or ATSec) at a given treatment (control or treated) and time condition (1 h or 4 h). Control was treated with DMSO for 1 h/4 h, while the treated condition included 1 µg/ml AmB for 1 h/4 h. Significance was calculated with a two-tailed paired *t*-test (*P < 0.05. **P < 0.01, ***P < 0.001, n.s.,not significant). The All Conditions category reaches a high significance level due to its larger sample size.

The program Salmon (25) was utilized to quantify the expressed genes. In WT, 65%–68% of clean RNA-sequencing (RNA-seq) reads could be mapped to transcripts of the Lodz University reference genome (26), while the rates were lower in ATSec (59%–66%) - a highly significant overall difference (*P* < 0.001)—indicating that ATSec is genetically more distant to the reference than WT. From these relatively low mapping rates, it follows that 32%–35% of WT reads and 34%–41% of ATSec reads could not be mapped to the reference (Fig. 4).

### A significant number of P-type ATPase genes are over-expressed

**Fig 5:**
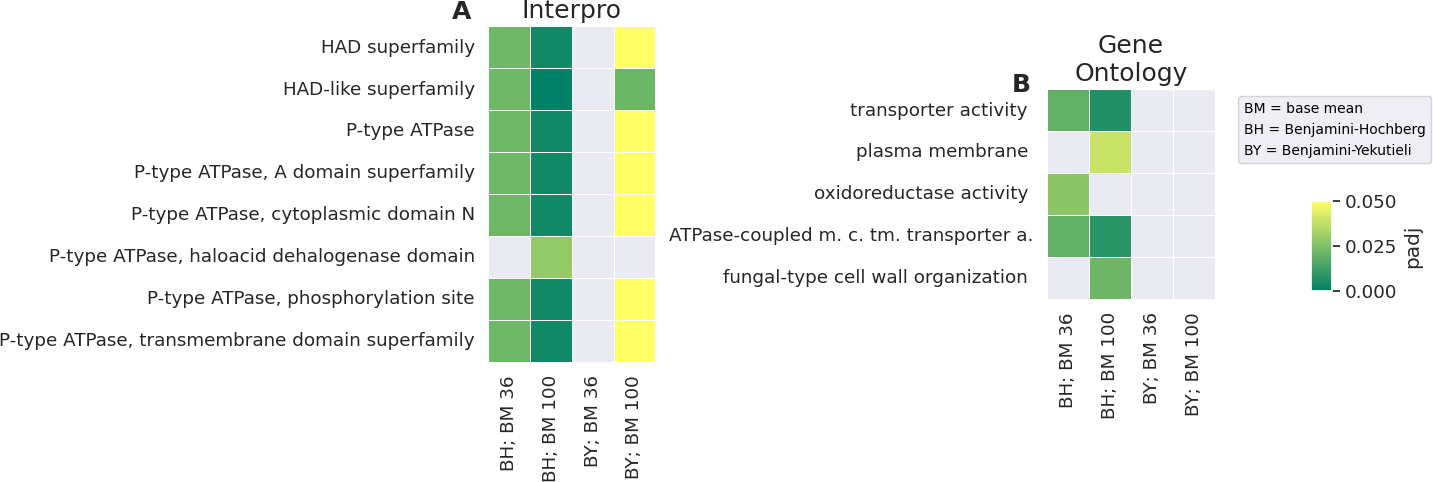
IDs enriched in over-expressed genes. Heatmap of all InterPro (**A**) or GO IDs (**B**) enriched in genes considered over-expressed at a BM cutoff of >36 (BM 36) or >100 (BM 100) with the BH or BY correction. IDs identified as enriched with the conservative BY correction are especially well supported since they are very unlikely to be false positives.

At the base mean (BM) >36 cutoff, 1,014 out of 10,744 genes—approximately 10%—were identified as differentially expressed between WT and ATSec in response to AmB. Of these, 731 genes were classified as over-expressed, showing a higher expression increase in WT compared to ATSec, while 283 genes were classified as under-expressed, with a lower expression increase (or even an expression decrease) in WT compared to ATSec. Typically, over-expressed genes exhibit their highest expression levels in treated WT (see Fig S2 and S4), whereas under-expressed genes display their highest expression levels in control WT (see Fig S3 and S5). When the BM cutoff was raised to >100, 871 genes or roughly 8% were identified as differentially expressed; 615 genes were over-expressed while 256 were under-expressed. Eight InterPro and five Gene Ontology (GO) IDs, were discovered to be significantly enriched (padj <0.05) in over-expressed genes in at least one of the conditions (Fig. 5). The InterPro IDs with the best support are the “HAD superfamily*”* and “HAD-like superfamily” as well as “P-type ATPase,” which are a subset of the “HAD superfamily,” and its various domains (Fig. 5). All genes carrying the ID “GO:0005215, transporter activity” or “GO:0019829, ATPases-coupled monoatomic cation transmembrane transporter activity” also carry the “P-type ATPase” ID (at BM >100 with the Benjamini–Hochberg [BH] correction). Furthermore, two-thirds of the over-expressed genes carrying the “P-type ATPase” ID also carry a “GO:0005886, plasma membrane” ID at BM >100 with the BH correction. The IDs “GO:0016491, oxidoreductase activity” and “GO:0031505, fungal-type cell wall organization” are enriched as well.

**Fig 6:**
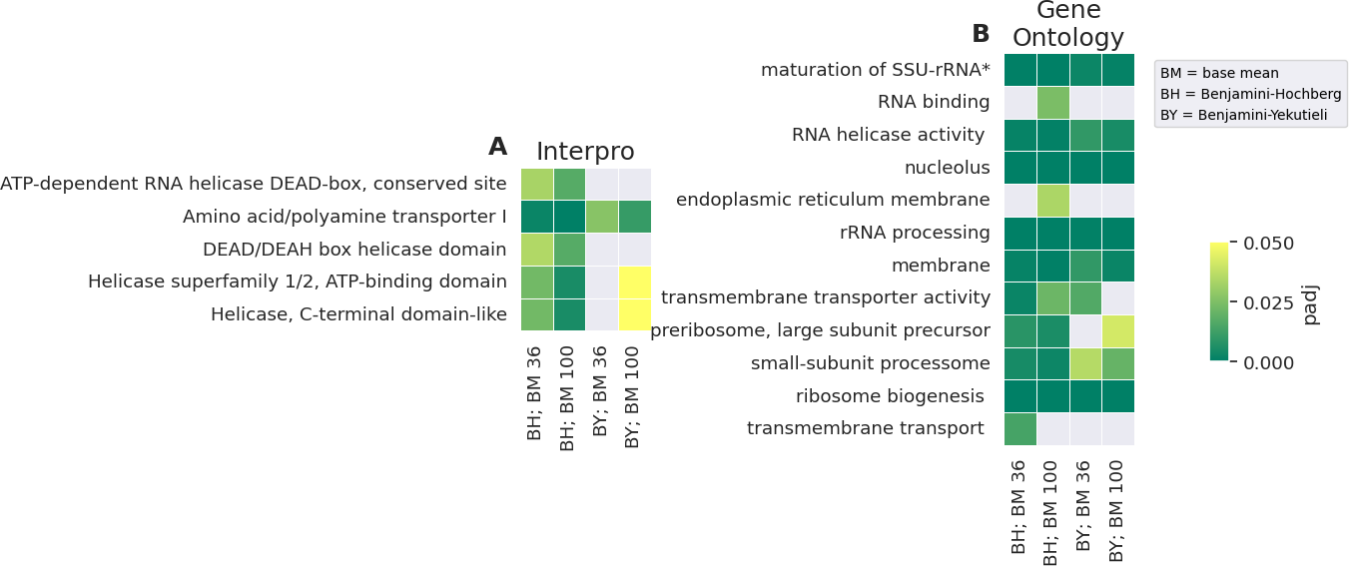
IDs enriched in under-expressed genes. Heatmap of all InterPro (**A**) or GO IDs (**B**) enriched in genes considered under-expressed at a BM cutoff of >36 (BM 36) or >100 (BM 100) with the BH or BY correction. IDs identified as enriched with the conservative BY correction are especially well supported since they are very unlikely to be false positives.

Five InterPro and 12 GO IDs, were discovered to be significantly enriched (padj <0.05) in under-expressed genes in at least one of the conditions (Fig. 6). Four of the enriched InterPro IDs relate to helicases, while the last one, “Amino acid/polyamine transporter I,” is an amino acid permease. Most of the best supported GO IDs relate to ribosomes, including “GO:0000462, maturation of small subunit rRNA,” “GO:0006364, rRNA processing,” “GO:0030687, preribosome, large subunit precursor,” “GO:0032040, small-subunit processome,” and “GO:0042254, ribosome biogenesis.” Additionally, “GO:0005730, nucleolus” and “GO:0005789, ER membrane” may also be ribosome related. Interestingly, no under-expressed gene carries more than three-ribosome connected annotations. GO terms related to RNA, such as “GO:0003723, RNA binding” and “GO:0003724, RNA helicase activity,” are also enriched. The latter ID shares a 100% overlap with the “DEAD/DEAH box helicase domain” InterPro ID in under-expressed genes.

Lastly, there are “GO:0022857, transmembrane transporter activity,” “GO:0055085, transmembrane transporter,” and “GO:0016020, membrane.” The first two are linked to all under-expressed genes associated with the “Amino acid/polyamine transporter I” ID.

### PKS connected to up-regulation and ATSec-exclusive mutations

Single nucleotide variants (SNVs) and structural variants (SVs) were identified. Around 4,800 genes carry a SNV at cutoff 0 while around 3,400 are identified at cutoff 50. SVs were much rarer with only 22 in 10,744 genes. Further details can be found in the Supplemental material.

**Fig 7:**
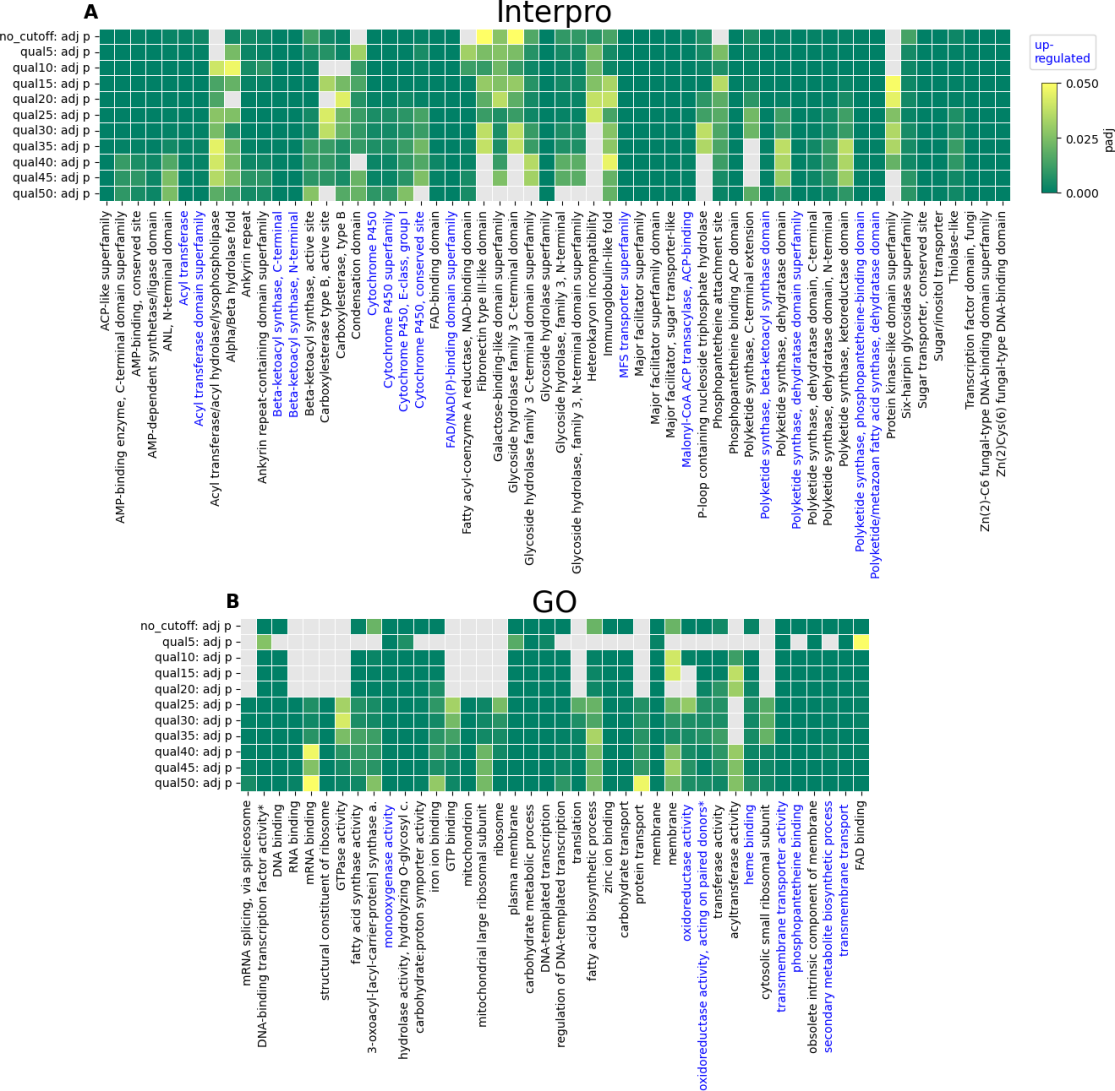
IDs with a significant enrichment of mutations. InterPro (**A**) or GO IDs (**B**) that were identified as mutated at six quality score cutoffs or more (corrected with the BH method) are shown. All IDs that were enriched in genes (corrected with the BH method) showing up-regulation (LFC >1, BM >36, p_adj_ <0.05) are highlighted in blue. They all exhibit well supported mutations, showing significant enrichment at the highest threshold tested. The vast majority of them was also identified as enriched at all quality thresholds. Two GO names (“maturation of SSU-rRNA from tricistronic rRNA transcript (SSU-rRNA, 5.8S rRNA, LSU-rRNA)” and “oxidoreductase activity, acting on paired donors, with incorporation or reduction of molecular oxygen”) were abbreviated in the figure. Some anomalous results are explained in the Supplemental material, ATSec-exclusive mutations, caveats.

Genes exhibiting up- or down-regulation between control WT and control ATSec as well as genes carrying mutations were identified separately. Of the genes with mutations significantly enriched at six or more quality score cutoffs, 15 InterPro and eight GO IDs were identified as significantly up-regulated in control WT compared to control ATSec (Fig. 7). Enrichment at high-quality scores is more meaningful because a higher-quality cutoff means the mutation call is more reliable. No ID carried a significant number of down-regulated genes (Fig. 7). The InterPro and GO IDs of genes showing up-regulation that also carry mutations (Fig. 7; Table 2) fit into three distinct categories, polyketide synthesis-related, cytochrome P450-related (CYP-related), and transport-related (Table 2). The one exception to this is “Rhodopsin domain, fungi,” which does not appear to fit any category.

**TABLE 2:** Gene categories up-regulated and mutated^*a*^

Category	IDs
Polyketide synthesis-related	Acyl transferase; acyl transferase domain superfamily; beta-ketoacyl synthase, C-terminal; beta-ketoacyl synthase, N-terminal; malonyl-CoA ACP transacylase, ACP-binding; PKS, beta-ketoacyl synthase domain; PKS, dehydratase domain superfamily; PKS, phosphopantetheine-binding domain; polyketide/metazoan fatty acid synthase, dehydratase domain; GO:0031177, phosphopantetheine binding; GO:0044550, secondary metabolite biosynthetic process
CYP-related	CYP; CYP superfamily; CYP, conserved site; CYP, E-class, group I; FAD/NAD(P)-binding domain superfamily; GO:0004497, monooxygenase activity; GO:0016491, oxidoreductase activity; GO:0016705, oxidoreductase activity, acting on paired donors, with incorporation or reduction of molecular oxygen; GO:0020037, heme binding
Transport-related	MFS transporter superfamily; GO:0022857, transmembrane transporter activity; GO:0055085, transmembrane transport

^*a*^ All InterPro and GO IDs (except for rhodopsin domain, fungi) that are enriched in genes that are mutated and also exhibit up-regulation have been put into three distinct categories based on the functional similarity of the IDs.

## DISCUSSION

While AmB resistance is generally rare among fungi, *A. terreus* exhibits relatively high resistance rates with 36.8% of isolates globally being resistant, with higher rates in Europe (40.1%) and Asia (40.4%) (27). Due to these facts, understanding the mechanism of AmB resistance in *A. terreus* is of particular interest. Previous studies have linked AmB resistance in different organisms to various mechanisms related to sterol composition (11), catalase activity (15, 18), cell wall content (17), and membrane composition (13, 20, 21). Our research group has previously reported that sectorization increases AmB susceptibility (10), leading to the current study attempting to uncover, which genes are differentially expressed in response to AmB treatment between WT and ATSec, thus uncovering genes potentially involved in AmB resistance/susceptibility that could serve as drug targets for novel therapies.

The primary finding of this project is that P-type ATPase genes are over-expressed in response to AmB treatment in WT. They exhibit the highest expression levels in treated WT, while the expression levels in control WT, control ATSec, and treated ATSec are lower (see Fig S2 and S4). This strongly suggests that the expression of these genes rises in response to the presence of AmB in resistant *A. terreus* and likely contributes to WT’s resistance. P-type ATPases constitute a large superfamily of ion pumps with various types having different substrate specificity (22, 28).

**Fig 8:**
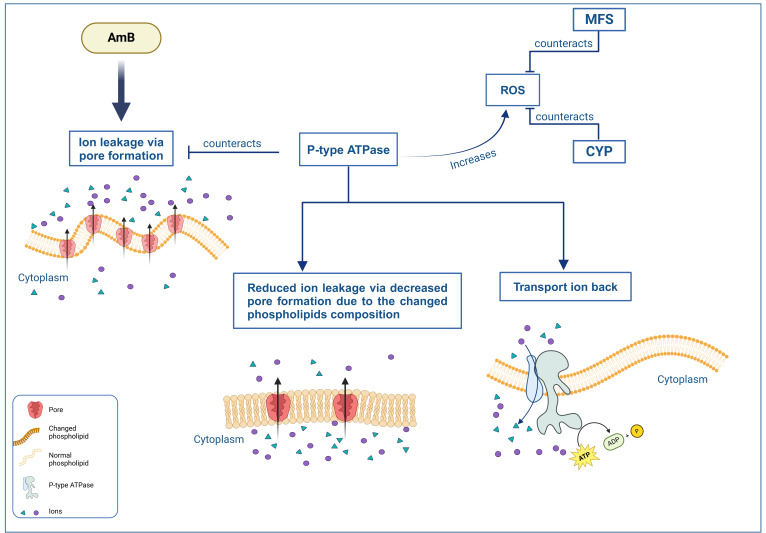
Proposed contributing factors to AmB resistance. AmB induces pore formation leading to ion leakage. P-type ATPases are proposed to counter this by changing membrane phospholipid composition, thus reducing pore formation, while also transporting leaked ions back into the cell, counteracting the leakage that does occur. P-type ATPases however deplete the cell of ATP and induce oxidative stress. MFS and CYP are known to counteract oxidative stress and might contribute to AmB resistance in that way. ROS; reactive oxygen species.

The question about the mechanistic basis of how P-type ATPases could contribute to AmB resistance still needs to be answered. It is known that AmB induces pore formation by interacting with ergosterol, leading to ion leakage and subsequent cell death (11, 13). Given their ion-transporting function (22, 28), P-type ATPases may mitigate this effect by transporting some of the leaked ions back into the cell (Fig. 8). Cohen describes such a reaction to AmB treatment as a futile cycle that will increase oxidative stress, ultimately leading to cell death (29). This suggests that for this mechanism to aid survival instead of hindering it, WT would need a way to reduce oxidative stress. Blatzer et al. (15) have provided compelling evidence for such a mechanism when they showed that an AmB-resistant isolate of *A. terreus* was more resistant to oxidative stress than a susceptible isolate and that increasing oxidative stress via prooxidants reduced AmB resistance.

A different potential resistance mechanism is related to the phospholipid membrane. It is known that P-type ATPases are involved in the transport of phospholipids to the outer membrane (21). Since a change in phospholipid composition is known to affect AmB-induced non-aqueous pore formation (14), this could also impact AmB resistance (Fig. 8). This hypothesis is supported by Fernandez-Prada et al. (21), who linked mutations in a P-type ATPase, specifically the miltefosine transporter, to AmB resistance in *Leishmania* (21). These same mutations are associated with a change in membrane composition (21), likely because of the miltefosine transporter’s direct involvement phospholipid translocation (30). Additionally, a change in membrane composition has also been linked to AmB resistance in *Kluyveromyces lactis* (20) and *Candida albicans* (13).

Therefore, it is plausible that P-type ATPases in *A. terreus* increase AmB resistance by altering the phospholipid content of the membrane. This would suggest a differential phospholipid composition between WT and ATSec, although that remains to be experimentally verified.

The second major finding highlights the role of PKS in sectorization (Fig. 7; Table 2). Our study finds that control WT exhibits up-regulation of polyketide synthesis genes compared to control ATSec. However, both genotypes have a similar expression change in response to AmB treatment. Polyketides are secondary metabolites, and it is well established that sectorized fungi often exhibit reduced production of secondary metabolites (1, 3). Up to now, however, no study found polyketides specifically being down-regulated in sectorized fungi. Polyketides have been reported to play are role in fungal virulence (31), with a prior study showing that sectorized *A. terreus* exhibited higher virulence than AmB-resistant *A. terreus* (10). In the current study, however, up-regulated polyketides do not correlate with virulence, as no significant difference in virulence between WT and ATSec was observed (Fig. 3), suggesting that the up-regulated polyketides are likely serving a different function.

Meng et al. (6) demonstrated that deletion of Pks11 results in defects in conidiation and alterations to the conidial cell wall structure in *Beauveria bassiana*. Furthermore, the deletion of Pks4 in *Trichoderma reesei* leads to the abolition of green pigmentation in conidia (7). In *Botrytis cinerea*, the deletion of Pks12 disrupts melanization of the sclerotia and leads to hypersensitivity to extreme temperatures and oxidative chemicals (8). Additionally, disruption of fluP, another PKS, eliminated asexual sporulation in *Aspergillus parasiticus* (9). Given that the phenotypic traits associated with sectorization, such as reduced pigmentation, defects in conidiation, and diminished sporulation (1, 3), show striking parallels to PKS mutations, we propose that sectorization is caused by mutations in genes involved in polyketide synthesis. Additionally, the influence of polyketides on oxidative stress resistance (8) may contribute to WT’s higher AmB resistance.

CYP-related genes are up-regulated in WT control strains compared to ATSec and carry ATSec-exclusive mutations (Fig. 7). Mutations in CYP-related genes have also been associated with phenotypic changes reminiscent of sectorized fungi. For instance, Shin et al. (32, 33) observed that some CYP mutants in *Fusarium graminearum* exhibited differences in pigmentation, conidiation, and increased susceptibility to azoles. Furthermore, there is a plausible link between these phenotypes and those resulting from mutations in polyketide synthesis-related genes, as certain CYP genes are involved in the synthesis of various polyketides, including lovastatin in *A. terreus* (34). Furthermore, an association between CYP genes and AmB resistance has been established. In *Leishmania donovani*, up-regulation of a CYP gene leads to AmB resistance due to altered sterol composition in the membrane (23). However, we did not observe any change in sterol composition (Table 1). Therefore, if CYP genes contribute AmB resistance in WT, they likely do so through a different mechanism or the composition change occurs only after the organism has been exposed to AmB. Fountain et al. (24) provide evidence that increased oxidative stress tolerance in *Aspergillus flavus* is connected to up-regulation of CYP. Given that AmB induces oxidative stress (11, 15, 18), CYP up-regulation might play a role in the higher AmB resistance of WT. It is even possible that this increased oxidative stress resistance is what allows the P-type ATPases to be over-expressed in treated WT without leading to oxidative death (Fig. 8).

Major facilitator superfamily (MFS) transporters are crucial for fungicide and oxidative stress resistance (35). Our study found that MFS transporters are up-regulated in control WT strains compared to ATSec and carry ATSec-exclusive mutations (Fig. 7). If MFS transporters can reduce oxidative stress and WT shows MFS up-regulation, WT should be less affected by AmB-induced oxidative stress (11, 15, 18), thus being more resistant against AmB, which appears to be true (Fig. 8).

Previous studies have documented both increased AmB susceptibility (36, 37) and resistance (19, 38) associated with mitochondrial mutations, underscoring the mitochondria’s role in AmB response. Consequently, it is plausible that the mutations identified in this study also influence AmB susceptibility or resistance. “GO:0005739, mitochondria” and “GO:0005762, mitochondrial large ribosomal subunit” show significant mutation enrichment at quality thresholds between 25 and 50 (Fig. 7), suggesting a notable proportion of these genes are mutated. Despite this, these genes are not differentially expressed (Fig. 5 and 6) nor do they exhibit up-regulation (Fig. 7). While many studies attribute differences in AmB response to changes in sterol levels (19, 37, 38), our findings show no difference in sterol levels (Table 1). Blatzer et al. (15) proposed an alternative mechanism involving mitochondrial ROS production, noting that AmB-susceptible *A. terreus* produce more ROS than resistant strains under AmB treatment. Further investigation is warranted, including comparing mitochondrial gene variations between other resistant and susceptible strains and testing if introducing mutations from resistant strains into susceptible ones can replicate the resistant phenotype, potentially establishing a genetic basis for AmB resistance testing.

In addition, the under-expression of ribosome-related genes in AmB-treated WT is notable (Fig. 6). These genes are most highly expressed in control WT, while control and treated ATSec exhibit intermediate expression levels (Fig S5). It has been previously reported that AmB causes down-regulation of ribosomal biogenesis genes in *Saccharomyces cerevisiae* (39). This suggests that the lack of down-regulation after AmB treatment in ATSec may be atypical, while the down-regulation in WT appears to represent a normal response to AmB.

An important limitation of our study is the low mapping rates observed in Salmon, which never exceed 68%. This issue has not been reported on in similar differential expression studies, where mapping rates are either not reported or are higher (40, 41). Some potential causes for these low mapping rates include contamination by adapter sequences, low-quality sequences, or high unknown nucleotide content, but these potential culprits were already filtered out with SOAPnuke (42), and FastQC (43) confirmed the success of the filtering. Another potential explanation, contamination by rRNA, was also ruled out based on low rRNA ratios reported by BGI (formerly Beijing Genomics Institute). Further analysis with Qualimap2 (44) indicated that only 61%–63% of reads from WT samples and 62%–65% from ATSec samples mapped to exons, while 12%–14% mapped to introns and 24%–26% (WT) or 23%–25% (ATSec) mapped to intergenic regions. This provides several potential explanations for the low mapping rate; the presence of intronic sequences is consistent with incompletely spliced RNA, novel splice variants, and/or genomic contamination; and the presence of intergenic regions is consistent with novel transcripts of unknown genes and/or genomic contamination. Salmon mapped 2.5–3.1 million reads per sample to the decoy (the whole-genome sequence), further supporting possible genomic contamination or unknown transcripts. An additional 3.1–3.9 million reads were discarded due to poor alignment scores and may stem from external contamination. FastQScreen (45) with Bowtie2 (46) was used to test for contamination from various organisms, including *Homo sapiens* and several fungi, with no significant contamination detected. However, 6%–13% of ATSec and 3%–5% of WT reads had no hits, suggesting possible contamination from unknown sources. This foreign contamination alone however cannot explain the low mapping rate by itself. Therefore, efforts to identify unknown transcripts or novel splice variants using StringTie were made. Unfortunately, this resulted in gene merging (neighboring genes getting assigned the same ID) and an increased multi-mapping percentage, negatively impacting the accuracy of quantification. Especially the gene merging was problematic since the expression levels of the merged genes get averaged meaning that an up-regulated gene and a down-regulated gene that were merged could appear as non-differentially expressed. Therefore, we chose to proceed with the low mapping rate rather than incorporate the additional issues introduced by StringTie (47). This however means that our analysis cannot make any claims about the expression levels of unmapped reads since those were not quantified.

### Conclusion

This study found strong evidence that P-type ATPases play a role in AmB resistance, thus enabling future research studying how the inhibition of these transporters may influence AmB resistance. Furthermore, we found evidence that mutations in polyketide synthesis pathways play a key role in sectorization. It remains to be seen if the predictions of this study such as a change in membrane composition between WT and ATSec will be experimentally confirmed, if over-expression of P-type ATPases can also be found in other AmB-resistant strains and if mutations and up-regulation of PKS and related genes are widely spread among other sectorized fungal strains. Future studies are expected to deepen our understanding of this phenotypic phenomenon and shed further light on the AmB resistance mechanism.

## MATERIALS AND METHODS

### Fungal strains

*A. terreus* WT isolate (Terrnet 211), identified previously (48) and cryopreserved in 10% glycerol at −80°C, was cultivated on SAB plate agar (BD Difco, USA) at 37°C for up to 12 days. Sector formation was assessed, and one of multiple spontaneously produced sectors (ATSec) was isolated as described previously (10), and sub-cultivated on fresh MEA (Carl Roth, Austria).

### Antifungal agent and AFST

Broth microdilution AFST for AmB (Sigma-Aldrich, A2411) against of WT and ATSec was performed following EUCAST guidelines E.Def.9.3.2 (www.EUCAST.org) (49).

### Sporulation assay

Sporulation of WT and ATSec isolates on ACM (50), AMM (50), and SAB agar plates was assessed as previously described (10). Briefly, spores were harvested by applying spore suspension buffer (0.9% NaCl, 0.01% Tween 20 [Sigma-P1379]), and spore solutions were filtered through 40 µm (PluriSelect Life Science, Germany) and counted with hemocytometer.

### *G. mellonella* virulence assay

The virulence assay was conducted using sixth instar larvae of *G. mellonella* (SAGIP, Italy), following the detailed procedure described previously (12). Fungal strains were grown on ACM agar (Carl Roth, Karlsruhe, Germany) for 5 days at 37°C. Spore suspensions were filtered through 40 µm (PluriSelect Life Science, Germany) and 5 µm (Sysmex, Germany) cell strainers, respectively, washed three times with insect physiological saline (IPS) (12) to remove hyphae and conidiophores. After counting with a hemocytometer, the suspension was adjusted to 10^8^ spores/ml in sterile IPS (12). Briefly, groups of 60 larvae were assigned to each of the following conditions, those injected with 20 µL of IPS, untouched larvae, and those infected with 2 × 10^6^ spores/larva of each WT and ATSec. The survival rate was monitored for up to 120 h at 37°C. Experiments were conducted in duplicates with different batches of larvae, each including three replicates (*n* = 60). The data from all experiments (120 larvae in total) were combined to calculate average survival rates every 24 h over the 120-h period.

### Sterol analysis

To obtain mycelia for sterol extraction, 10^6^ spores of WT and ATSec strains of *A. terreus* were cultivated in 250-mL Erlenmeyer flasks containing 100-mL Sabouraud dextrose broth (BD Difco, USA) (10^6^ spores per flask) at 30°C with shaking at 180 rpm overnight (16 h). The cultures were afterward harvested by filtration, washed with distilled water, and freeze-dried to quantify the fungal biomass on a dry weight basis. For sterol extraction, 3 mg of lyophilized mycelia were homogenized and sterols isolated as previously described (51). Relative and absolute amount of sterols (11 different sterols) of two technical replicas of two biological samples were determined by GC-MS according to Müller et al. (52).

### RNA-sequencing

WT and ATSec strains were grown in AMM broth for 30 h at 30°C. Afterward, the cultures were subjected to either DMSO (control) or 1-µg/mL AmB treatment for 1 h/4 h. Triplicates of each combination of these factors were made, resulting in a total of 24 samples. RNA was extracted with a Promega GmbH kit (Madison, Wisconsin, USA) following the manufacturer’s instructions. Library construction was conducted by BGI using reverse transcriptase and random primers to generate cDNA which then had adapters ligated to it and was amplified via PCR. The yield was quantified by Qubit, and ssDNA circles were constructed. With the DNA nanoball sequencing (DNBSEQ) platform, a total of around 20 million 100-bp paired-end reads were generated. BGI removed reads mapped to rRNA and employed SOAPnuke (version 1.5.2 with the settings -n 0.001 L 20 -q 0.4 A 0.25 -Q 2 G) (42) to remove adaptor-containing sequences, low-quality reads, and reads with high unknown nucleotide content, producing “clean reads” data sets that were used in the analysis. On average, clean read data sets contain 45.1 Mb of reads, while the raw read data sets contain 48.7 Mb. For clean reads, the mean Q20 value is 98.3%, and the mean Q30 value is 92.5%.

### Nanopore genome sequencing

The genome of WT and ATSec were sequenced with Oxford Nanopore Technologies (ONT). Guppy (v. 4.2; ONT) was used for base calling, adapter removal, and quality verification. The two nanopore read bundles were then assembled using the Flye assembler (53) in Galaxy (v. Galaxy Version 2.6) utilizing the “Nanopore Corrected” mode and the “35 m” genome size setting. The Quast assembly quality tool (54) in Galaxy (v. Galaxy Version 5.0.2+galaxy1) was used to determine the quality of the assemblies. The estimated genome size setting was “35,000,000,” and the organism type was “Fungus.” Any options not specified were left at default. Data can be found on the National Center for Biotechnology Information (NCBI) Sequence Read Archive (SRA) under the accession PRJNA1161802 (55). Samtools (56) calculates a coverage of 94% (WT)/95% (ATSec) and a depth of 94.0 (WT)/112.8 (ATSec) based on alignments generated with minimap2 (57).

### Coding and graphs

R versions 4.3.1 (16 June 2023) and 4.3.2 (31 October 2023) (58) were used to analyze differential gene expression employing DESeq2 (59), tximport (60), and ashr (61),

Python (version 3.11.8) (62) was used to write custom scripts for enrichment analysis and figure creation (Fig. 5 to 7), employing the following Python libraries: Bio, collections, csv, gzip, io, itertoolsm mpl_toolkits, Numpy, os, re, scipy, Seaborn, statsmodels, tkinter, and vcf. Figures 2 to 4 were created with GraphPad Prism (version 10.2.3), and Fig. 8 was created in BioRender, Vahedi, R. (2025) https://BioRender.com/o36y907.

Jupyter-Lab (version 3.5.3) (63) was the development environment for python and R scripts. Anaconda (conda version 24.5.0) is a distribution of Python and R, specifically designed for scientific computing (64). The Anaconda Navigator was used to launch the development environment, and the conda command was employed to install packages.

### HISAT2

HISAT2 (version 2.2.1) (65) was used to align RNA-seq reads to the genome assembly ASM1680841v1 by the Lodz University of Technology (26). The output was sorted with *samtools sort* (56).

### Salmon

The program Salmon (version 1.10.1) was used to map and quantify the RNA-seq reads against the Lodz reference (25). Salmon was run in quasi-mapping read mode, with the whole genome as a decoy and with the validateMappings option enabled (25).

### StringTie

StringTie (version 2.1.7) was used in an attempt to assemble transcripts (47). For the initial run, the read depth was set to 1.5, the minimum isoform abundance to 0.05, and the minimum transcript length to 300 with the other settings remaining at default. For the merge run, the minimum transcript length was set to 300 and the minimum FPKM to one with the other settings at default.

### False discovery rate correction

In the InterPro and GO ID enrichment analysis, BH (66) and the Benjamini–Yekutieli (BY) (67) were used for false discovery rate correction.

### DESeq2

The differential gene expression analysis with DESeq2 (version 1.44.0) (59) was performed on the gene level rather than the transcript level. The analysis identified the difference of the treatment effect between WT and ATSec. NaN values were filtered out of the results, and the ashr shrinkage method (61) was applied. Cutoffs for counting a gene as differentially expressed were set as described by Hokken et al. (68). However, a more conservative adjusted *P*-value cutoff (<0.05 instead of <0.1) was set and in addition, and the BM cutoff was set at >36 and >100. A second analysis was performed comparing only expression levels of control WT and control ATSec. The cutoffs were |LFC| > 1, p_adj_ <0.05, BM >36.

### InterProscan

InterProscan (version 5.66–98.0) (69) was used to generate InterPro and GO annotations for all genes in the reference transcriptome. The nucleotide flag was set, and the transcriptome fasta file, which was generated with *gffread* (70), was split into chunks using *seqkit split* (71). The output was then merged using the commandline *cat* command. Data used in our analysis can be found in File S2.

### Mutations

ATSec-exclusive SVs were identified in Oxford Nanopore data using minimap2 (57), *samtools sort* (56), Sniffles2 (72), *bedtools subtract*, and *bedtools intersect* (73).

ATSec exclusive SNVs were identified in RNA-seq based HISAT2 (65) alignments using *bcftools mpileup* (56) with the read depth set to 1 million, *bcftools call*, and *bcftools filter* (56), with quality score thresholds between 0 and 50 as well as *bedtools subtract* (73) and *bedtools intersect* (73).

### Quality control

FastQC (version 0.12.1) (43), Qualimap2 (version 2.3) (44), and FastQscreen (version 0.15.3) (45) were used for quality control of the RNA-seq data. FastQscreen was used with Bowtie2 (46) indices built with the genomic data of *H.sapiens (GRCh38), Escherichia coli (GCA001606525.ASM160652v1), Arabidopsis thaliana (TAIR10), Caenorhabditis elegans (WBcel235), S. cerevisiae (R64-1-1), C. albicans 19F (GCA_000775445), Aspergillus fumigatus (ASM265v1*), and *A. niger (ASM285v2*) taken from the Ensembl database (74).

## Data Availability

The nanopore sequencing read files can be found on the NCBI SRA under the accession PRJNA1161802 and the RNA-seq data on the Gene Expression Omnibus (GEO) under the accession GSE277670.

## SUPPLEMENTAL MATERIAL

File S1 mbio.03926-24-s0001.docx Supplemental figures and a more detailed description of the methodology.

File S2 mbio.03926-24-s0002.csv InterPro and GO annotations found using InterProScan.

File S3 mbio.03926-24-s0003.xlsx Numerical data used in table and figures.

Supplemental legends mbio.03926-24-s0004.txt Legends for Files S2 and S3.

ASM does not own the copyrights to Supplemental Material that may be linked to, or accessed through, an article. The authors have granted ASM a non-exclusive, world-wide license to publish the Supplemental Material files. Please contact the corresponding author directly for reuse.
