# Supplementary material for: Aspergillus terreus sectorization: a morphological phenomenon shedding light on amphotericin B resistance mechanism
Source: mBio. 2025 Feb 25;16(4):e03926-24. doi: 10.1128/mbio.03926-24 (PMC11980562; doi:10.1128/mbio.03926-24)
Supplement: File S1 — Supplemental figures and a more detailed description of the methodology. [file mbio.03926-24-s0001.docx]

# Supplemental Material

## Single Nucleotides Variants are More Common Mutations than Structural Variants

Two types of mutations were identified at different quality score cutoffs (Sup 1): The more abundant single nucleotide variants (SNVs) and the much rarer structural variants (SVs). The quality score is crucial as genes with a higher quality score are also more likely to be genuinely mutated rather than just incorrectly identified as such. Thus, Sup 1 shows how increasing the confidence in a mutation call initially reduces the number of genes where at least one ATSec-exclusive mutation is being called before the number stabilizes around 32%. Only 22 of 10744 genes had a SV, accounting for less than 1% of mutated genes, but all SVs had a quality score >50. Around 4800 genes carry a SNV at cutoff 0 while around 3400 are identified at cutoff 50.


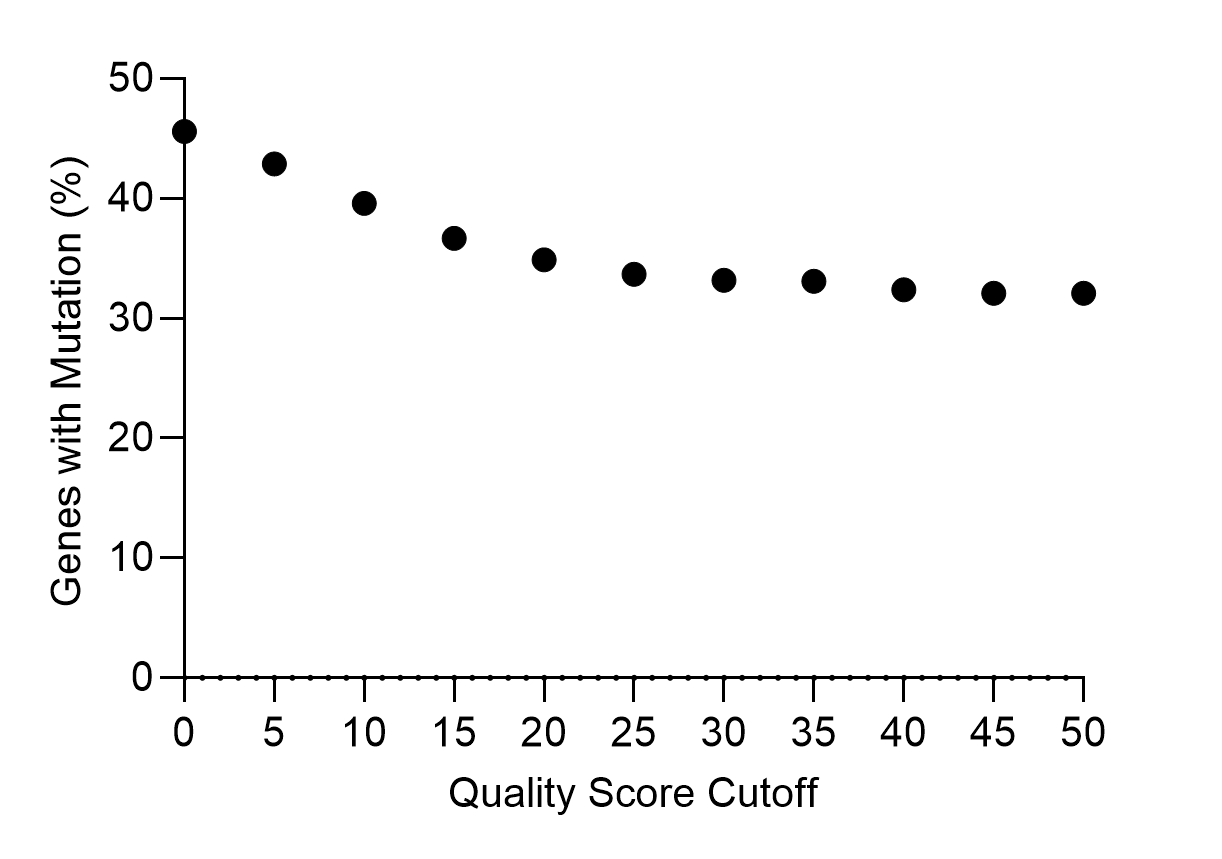


**S1 Fig 1. Mutation Percentage**. Percentage of genes carrying an ATSec-exclusive mutation (single nucleotide variant or structural variant) at a given quality score cutoff.

Of the 22 genes containing a SV unique to ATSec, 14 possess GO annotations and 17 have InterPro annotations. No ID type is significantly enriched in the genes with SVs. However, 1 Cytochrome P450 gene and 1 Polyketide synthase gene are disrupted by a ATSec-exclusive SV.

## ATSec-exclusive mutations: Caveats

The results in Figure 7 do accurately reflect the finding of genes with at least one mutation call unique to ATSec. There is however a seeming randomness to some of the results in – for example “carboxylesterase type B”, “immunoglobulin-like fold” and in “GO:0003429: mRNA binding”. These unusual results are a consequence of only counting ATSec exclusive mutations. The problem can be illustrated as follows: We detect low quality mutation call at locus A and a high quality mutation call at locus B of the same gene in ATSec in combination with an intermediate quality mutation call at locus A in wildtype. At a low cutoff, the high quality mutation call in ATSec is disregarded due to the corresponding mutation call also being present in wildtype. The low quality mutation call of the same gene is however still detected. When the cutoff is now raised to an intermediate level, the low quality mutation call of ATSec is filtered out but the intermediate mutation call of wildtype is not. Thus no mutation unique to ATSec is called. When the cutoff is now raised to a high level, the intermediate wildtype mutation call is filtered out and only the high quality mutation call remains.


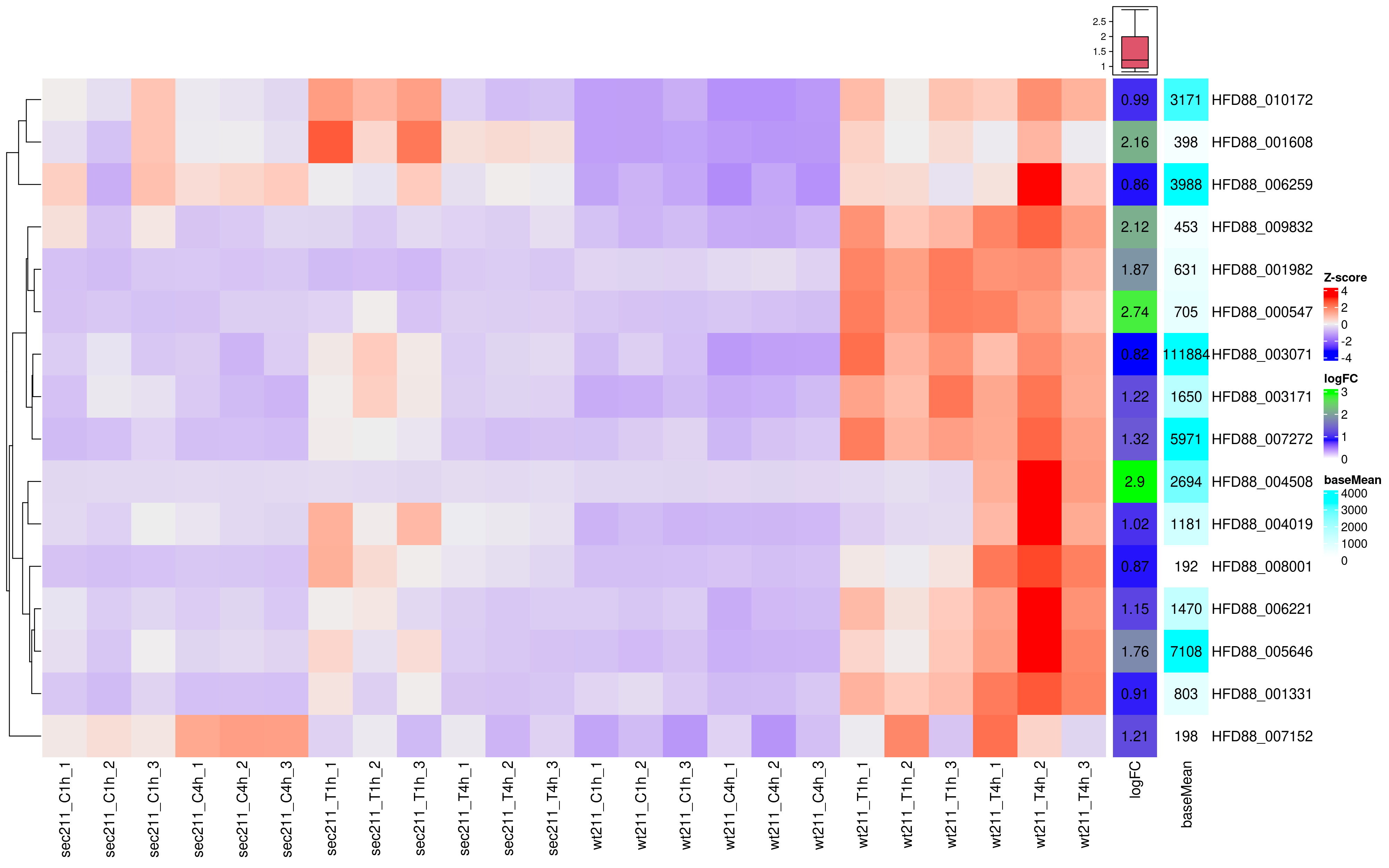
**S1 Fig 2. Over-expressed Genes (InterPro).** Genes with p_adj(BH)_<0.05, LFC>0.7, BM>100 and contributing to the enrichment of at least 25% of InterPro IDs significantly enriched in over-expressed genes.


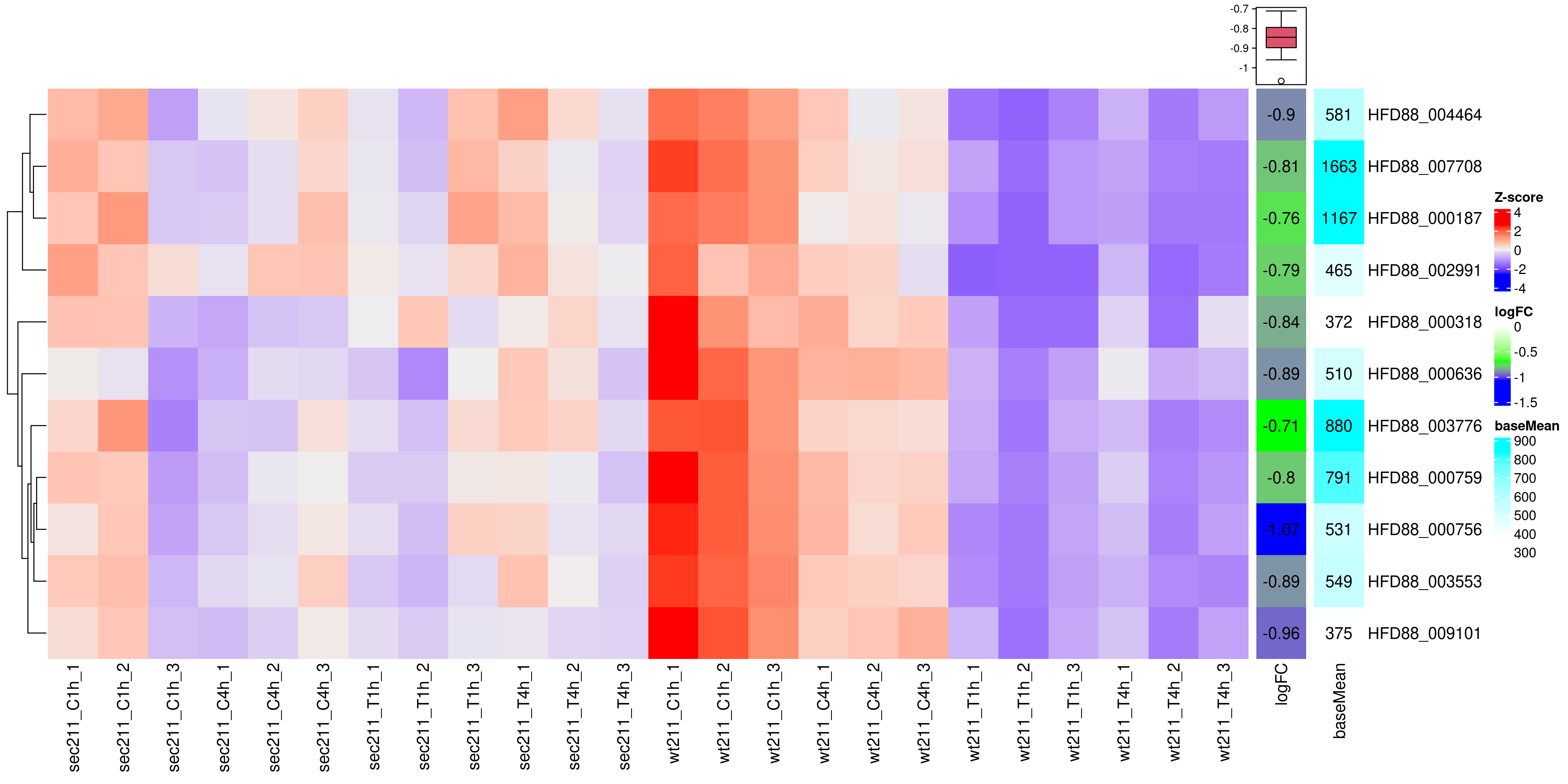
**S1 Fig 3. Under-expressed Genes (Inter Pro).** Genes with p_adj(BH)_<0.05, |LFC|>0.7, BM>100 and contributing to the enrichment of at least 25% of InterPro IDs significantly enriched in under-expressed genes.


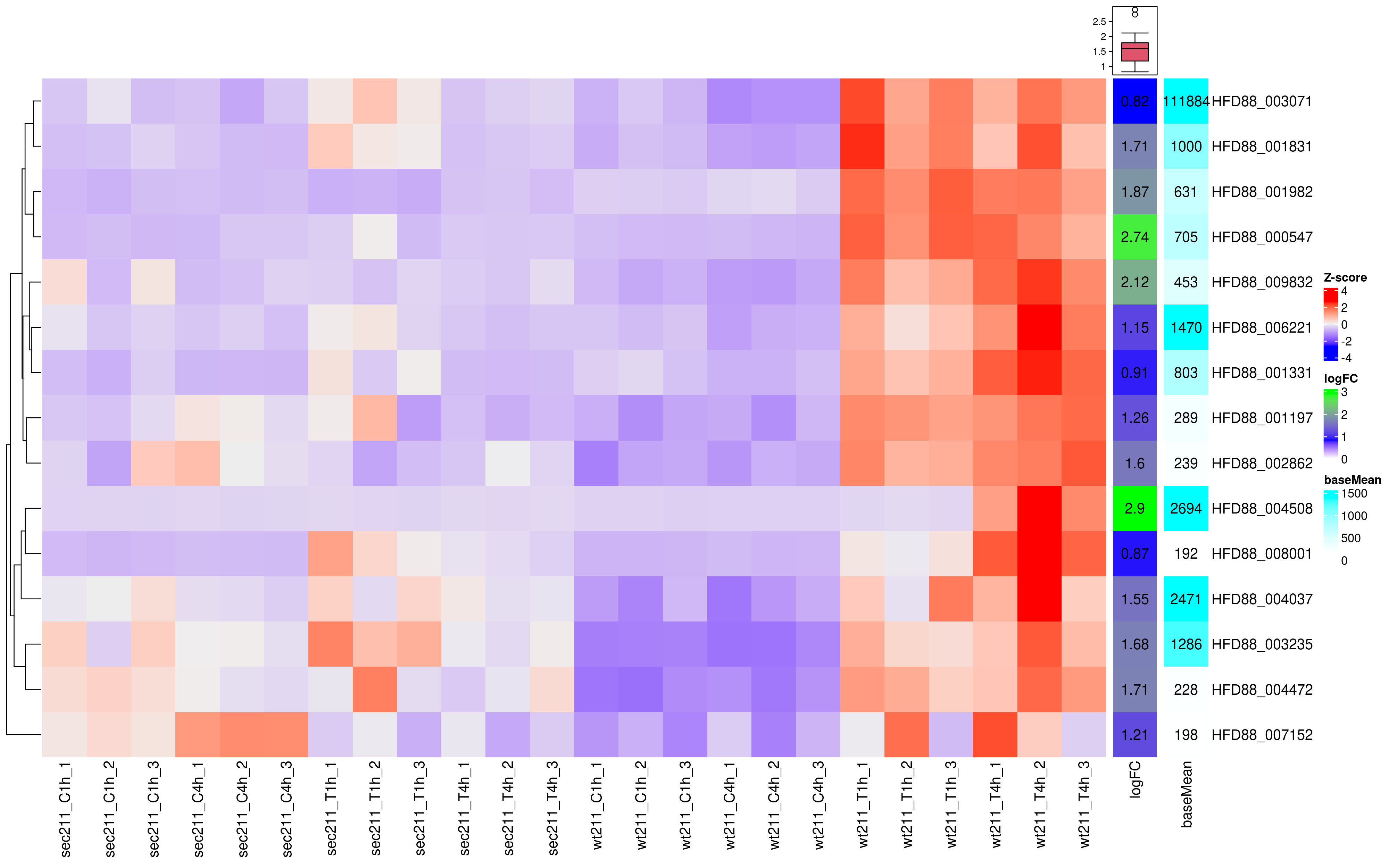
**S1 Fig 4. Over-expressed genes (Gene Ontology).** Genes with p_adj(BH)_<0.05, LFC>0.7, BM>100 and contributing to the enrichment of at least 25% of Gene Ontology IDs significantly enriched in over-expressed genes.


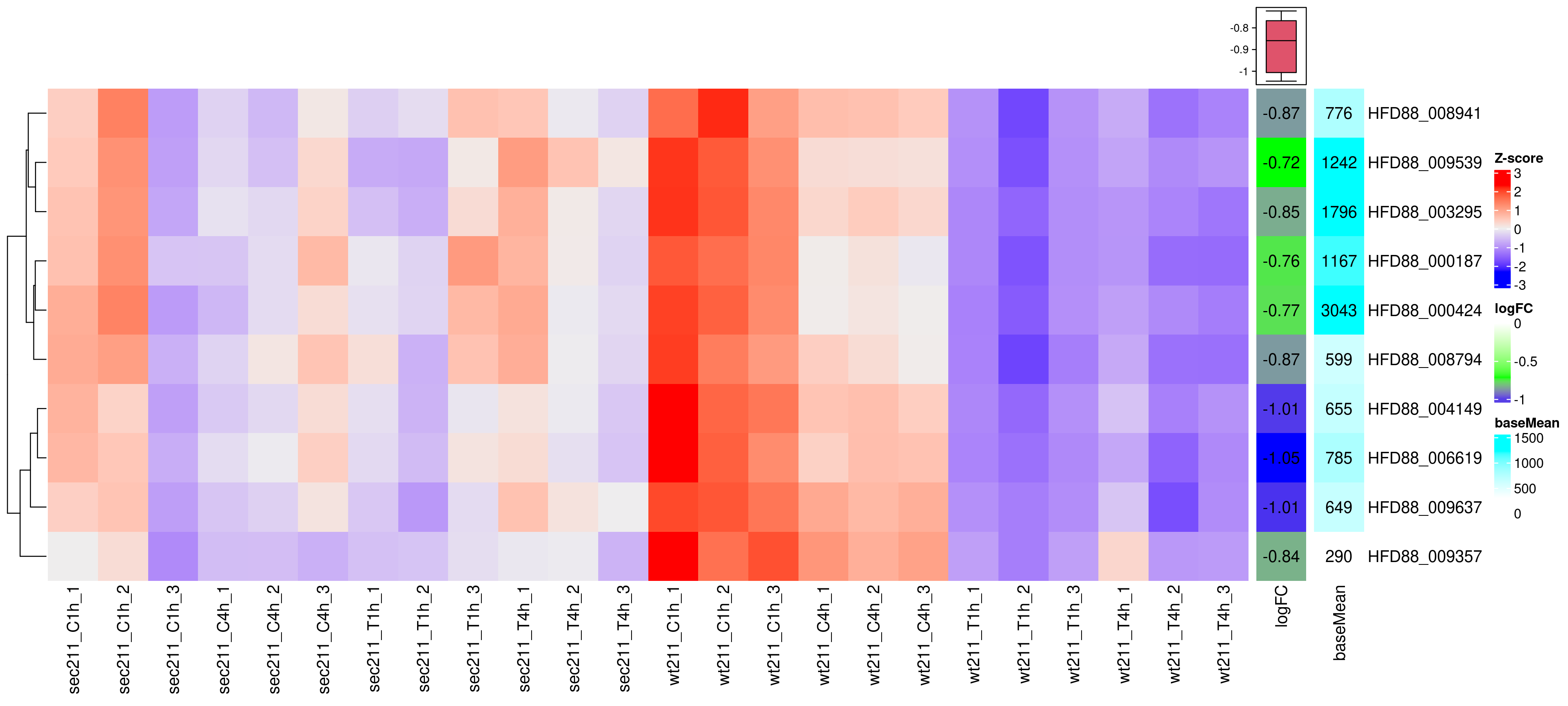
**S1 Fig 5. Under-expressed genes (Gene Ontology).** Genes with p_adj(BH)_<0.05, LFC<-0.7, BM>100, contributing to the enrichment of at least 25% of Gene Ontology IDs significantly enriched in under-expressed genes.

# Detailed Materials and Methods

## Hardware

A HP Z2 G9 desktop with an Intel core i9 13900K CPU and 32GB of DDR5 RAM was used for the data analyis. The i9 13900K is a high-end consumer-grade Intel CPU of the 13^th^ generation. It has 8 p-cores (performance cores) with 2 threads each as well as 16 e-cores (efficience cores) with 1 thread each, giving it a total of 32 threads (1). The memory of the PC is a single channel 4800Mhz module. This is inferior to a dual channel configuration (2 memory modules installed in two different channels) that would greatly improve the memory bandwith by having two RAM modules accessible simultaneously. During this project, both the CPU, motherboard and CPU cooler were swapped out by service technicians due to overheating issues of the PC. The modifications did not fix the overheating issue which had negative effects on the PCs performance as cores have to reduce clock speed once a critical temperature is reached to protect the components from permanent damage due to overheating. This safety mechanism is called “thermal throttling”. Despite thermal throttling, the maximum operating temperature of 100°C was exceeded several times according to HWinfo (v7.72-5355) reports (2). There was also no option to add additional fans to the case to provide additional cooling as there was only a single, already occupied, mounting point for a standard 80mm fan and no mounting points for 120mm or 140mm fans. Furthermore, the BIOS of the machine either did not offer an adjustable fan curve nor the option to reduce the power draw of CPU or the technicians did not make use of these functions.

After the completion of the data analysis, Intel confirmed on their website, that elevated operating voltage causes instability in their 13^th^ and 14^th^ generation desktop CPUs. Elevated operating voltage can also cause spikes in core temperature or even physically damage a CPU. A microcode patch to fix the issue was also announced (3).

## Virtual machine

Since the HP Z2 desktop is a Windows machine, all Linux based programs were run with Oracle VM Virtual Machine 7.0 (4) in Ubuntu 22.04.3 LTS. This virtual machine had 23 threads and 23.6 GB of memory allocated to it because allocating more to it would have risked making the Windows host system unstable and less responsive. All software mentioned below was run on this virtual machine unless specified otherwise.

## R

R is a programming language commonly used for scientific applications (5). During this work, R versions 4.3.1 (2023-06-16) and 4.3.2 (2023-10-31) were used to analyze differential gene expression with the libraries DESeq2 (6) and tximport (7). For the creation of heatmaps (Sup 2-5), the libraries gplots (8), grid (5), ComplexHeatmap (9, 10) and RcolorBrewer (11) were used. Furthermore, ashr (12) was used for log2FoldChange shrinkage.

## Python

Python is another programming language commonly used in scientific projectes (13). In this project version 3.11.8 was used and custom programs for graph plotting and enrichment analysis were written using this coding language. The python libraries used in the scripts written for this project are provided in the table below.

| Python libraries | | | | |
| --- | --- | --- | --- | --- |
| Bio (14) | collections | csv (15) | glob | gtfparse (16) |
| gzip | io | itertools | math | matplotlib (17) |
| mpl_toolkits | Numpy (18) | os | Pandas (19) | pyranges (20) |
| re | scipy (21) | Seaborn (22) | scikit-learn (23) | statistics |
| statsmodels (24) | tkinter | vcf |  |  |

## Jupyter-Lab

Jupyter-Lab (version 3.5.3) is a development environment that natively supports python scripts and can be made to support R scripts as well by simply setting up the R kernel (25). It was the primary development environment used in this project.

## Anaconda

Anaconda (conda version 24.5.0) is a distribution of Python and R and is specifically designed for scientific computing (26). The Anaconda Navigator offers easy access to many developlment environments like JupyterLab, Rstudio and Pycharm. It also includes the conda package manager which will check for dependency conflicts which other package managers like pip do not. In this study, it was primarily used to install python packages and launch JupyterLab with the Anaconda Navigator*.*

## Graph Prism

Figure 2-4 were created with GraphPad Prism (version 10.2.3), an illustration software with a subscription-based monetization model. It was run on Windows since no Linux version exists and the developers have no plans of releasing one in the future but give advice on how it may run on Linux using Wine (27).

## Nanopore genome sequencing

The genome of wildtype strain 211 and ATSec strain 211 were sequenced with Oxford Nanopore Technologies (ONT). The long read sequencer minION was used, which has the advantage of creating long reads thus keeping genes intact. This however comes at the cost of the method being more error-prone and leading to a lower sequence quality. The raw nanopore data was base called by Westerdijk Fungal Biodiversity Institute using Guppy (v. 4.2; Oxford Nanopore Technologies, Oxford, United Kingdom). Guppy also removed adapters and verified the quality of the sequences using Phred scores. The generated fastq files were uploaded to Avans’ secured Midgard and Galaxy server. Any time galaxy is mentioned, this refers to Avans’ secured private server. The 2 nanopore read bundles were then assembled using the Flye assembler (28) in Galaxy (v. Galaxy Version 2.6). The Flye assembler can tolerate a higher error level in sequences due to its use of repeat graphs, thus being a good choice for our purpose. For the mode option, ‘Nanopore Corrected’ was chosen and for the estimated genome size ‘35m’ was entered. The other options were left at their defaults. After the assembly, the Quast assembly quality tool (29) in Galaxy (v. Galaxy Version 5.0.2+galaxy1) was used to determine the quality of the assemblies. The estimated genome size was set to ’35,000,000’ and the type of organism was set to ‘Fungus’. The rest of the options were left at their defaults.

## HISAT2

HISAT2 (version 2.2.1) is a splice aware aligner meaning it can align RNA-seq data to a reference genome by accounting for the intron(s) that a multi-exon read contains, which are present in the genome but missing from the RNA data (30). HISAT2 was developed for the human genome and its variants (30). The reference genome the reads were aligned to in this analysis was the *A. terreus* genome assembly ASM1680841v1 by the Lodz University of Technology (31). This assembly was chosen over the assembly ASM14961v1 by the Broad Institute due to the Lodz assembly being more recent (Lodz was submitted in 2021, the Broad Institute assembly in 2006) and containing more genes and protein coding sequences. Like the Broad Institute, the Lodz University assembly is haploid and has scaffolds instead of assembled chromosomes. One difference is that the Lodz genome has 9 scaffolds (according to the NHI database) or 8 contigs and 1 scaffold (according to the terminology used in the paper (31)) while the Broad Institute’s has 26 scaffolds.

The sam files generated by HISAT2 were converted into bam files and sorted with *samtools sort* (32)*.*

## Salmon

Salmon (version 1.10.1) was used to map and quantify the RNA-seq reads against the Lodz reference because it is widely known for being a fast and accurate tool (33). Salmon was run in quasi-mapping read mode, meaning that the analysis was run with the clean read fastq files themselves rather than aligned sequences. Salmon accepts both fastq files and unsorted alignments in bam format as valid input for according to its documentation (33). The analysis was run with the whole genome as a decoy meaning that all reads that map better to the genome than to the transcriptome are filtered out and assumed to be DNA contamination of the RNA-seq data. Furthermore, the validateMappings option was enabled. The validateMappings option improves sensitivity by employing minimap2’s chaining algorithm (33, 34) for scoring, mapping loci and the dynamic programming algorithm SIMD from ksw2 (35) for scoring and validation.

## StringTie

StringTie (version 2.1.7) is a transcript assembly program (36). It requires a reference genome for transcriptome assembly and uses concepts from de novo genome assembly to improve the accuracy of the assembled transcript (36). Steps of the Tuxedo protocol from the StringTie manual were followed (37): A .gtf file for every RNA-seq experiment was constructed using the corresponding bam file created with HISAT2 and the .gff file of the reference genome. The read depth was set to 1.5, the minimum isoform abundance to 0.05 and the minimum transcript length to 300. Following that step, the resulting .gtf files were merged using the --merge function. The minimum transcript length was kept at 300 and a minimum FPKM of 1 was set. The generated merged gtf file was then used as the reference for the final step and the reads were quantified on the basis of the new reference.

### False Discovery Rate Correction

For false discovery rate correction for the Interpro ID and Gene Ontology enrichment analysis, the Benjamini-Hochberg (38) and the Benjamini-Yekutieli (39) method were used. The Benjamini-Hochberg method is more commonly used because it has a higher power, it does however rely on the assumption that there is either no dependence or a positive dependence between values. The Benjamini-Yekutieli method on the other hand is more conservative (meaning it will make fewer false positive calls but will usually make more false negative calls) but does not make that assumption.

## DESeq2

DESeq2 (version 1.44.0) is an R package which is available through the Bioconductor repository (6). It enables the detection of differentially expressed genes and accepts input from the quantification tools Salmon, RSEM, kallisto, Sailfish, Alevin and StringTie. In this study, tximport (7) was used to import the Salmon results into DESeq2. The differential gene expression analysis was performed on the gene level rather than the transcript level meaning that – if a gene had multiple transcripts – they were counted together. A design of genotype, treatment, time and an the interaction term genotype:treatment was used. The interaction term allows us to see the difference of the treatment effect between wildtype and ATSec, with the wildtype being the reference level and a detailed explanation can be found in the DESeq2 vignette (40).

In our study, design matrix distinguishing 2 genotypes (wildtype and ATSec), 2 conditions (control and treated) and 2 times (1h and 4h) was provided to DESeq2 (6). An interaction term allowing us to see the difference of the treatment effect between wildtype and ATSec, with the control wildtype being the reference level was introduced. NaN values were filtered out of the results and the ashr shrinkage method (12) included in the package was applied. This was done because the creators of DESeq2 recommend the use of a shrinkage procedure as a way to improve dispersion estimates and avoid false positives when identifying differentially expressed genes (6) – in this case genes who react differently to treatment in wildtype and ATSec. After the shrinkage procedure, cutoffs for counting a gene as differentially expressed were set to an a log2fold change (LFC) >0.7 or <-0.7 (also referred to as |LFC|>0.7) and a base mean (BM) >36 since those were the cutoffs used by Hokken et al (41) in a similar study investigating azole resistance in *A. fumigatus*. Hokken et al however used STAR instead of Salmon for the quantification of genes and had a higher mapping rate. This study used a more conservative adjusted p-value cutoff (<0.05) than Hokken et al used (<0.1). A second analysis was also performed where all cutoffs were kept identical except for BM which was raised to >100.

In addition, a seperate DESeq2 analysis was performed comparing only expression levels of control wildtype and control ATSec. A design matrix only distinguishing genotype and time was used because there were no different treatment conditions. This analysis was meant to find genes that were differentially expressed between wildytpe and ATSec without the influence of AmB which would hint at genes . The cutoffs were slightly change to the prior analysis (|LFC|>1, p_adj_<0.05, BM > 36).

## Interproscan

Interproscan (version 5.66-98.0) is a program for identifying protein domains based on their sequence and their pattern/signature which allows prediction of a protein’s function (42). The program is available as a web version or as a standalone command line version. The command line version can handle larger datasets and was thus used in this project instead of the webtool. Due to the fact that a nucleotide sequence in the form of the reference transcriptome and not a protein sequence were provided, the nucleotide flag had to be set (42). To improve performance, the transcriptome fasta file, which was generated with *gffread* (43) from the genome fasta file and a gff/gtf file containing the transcripts, was split into chunks using *seqkit split* (44). The .tsv output of those chunks was then merged again using the command line *cat* command. Annotations from all databases (CATH-Gene3D, CDD, HAMAP, NCBIfam, PANTHER, Pfam, PIRSF, PRINTS, PROSITE profiles, PROSITE patterns, SFLD, SMART and SUPERFAMILY) as well as associated Gene Ontology terms were generated (42) but only annotations that also had an Interpro ID were considered. This was done to avoid similar/synonymous annotations from different databases being treated as seperate variables.

### Structural variants

Structural variants (SVs) were identified from genomic data of the strains generated with Oxford Nanopore. First, the Oxford Nanopore reads were aligned to the reference genome with minimap2 (34, 45). The minimap2 *map-ont* option was used due to it being specifically designed for Oxford Nanopore reads. The program also offers a setting optimized for Illumina Complete Long Reads and another for Pacbio CLR reads. The sam alignment files of both WT and ATSec were processed with *samtools sort* (32) and converted into bam format to save storage space. Sniffles2 (46, 47) was then used to analyze the sorted bam files for SVs. In the next step, *bedtools subtract* (48) was used to remove all SV that ATSec shared with wildtype so that only the ATSec-exclusive mutations remained. In the final step, *bedtools intersect* (48) was used to indentify all genes that contained at least one ATSec-exclusive SV. SV outside of genes were not further analyzed.

### Single nucleotide variants

To identify SNVs, all RNA-seq based HISAT2 (30) alignment files of a genotype (from all conditions) were processed with *bcftools mpileup* (32) together with the reference genome. Bcftools (32) is a program for variant calling and for the manipulation of variant calling files. Because the default read depth was low, it was increased to an extremely high value of 1 million to ensure that all reads for a position would be taken into account at the cost of requiring more processing power and memory. This generated genotype likelihoods that were then provided to *bcftools call* (32) which was using the default calling method and returned the variant sites. *Bcftools call* however allows variants with low quality score (a metric for how certain we can be that the detected SNV is actually there) to pass because there is no quality filter which means without setting a minimum quality score. Using *bcftools filter* (32), a filtering step could be performed. Quality thresholds from 0-50 (in steps of 5) were used to test which was best suited.

The ATSec and wildtype file of each threshold were again processed with *bedtools subtract* (48) to remove all SNV that ATSec shared with wildtype so that only the ATSec-exclusive mutations remained. Following that, *bedtools intersect* was used to indentify all genes that contained at least one ATSec-exclusive SNV. SNV outside of genes were not further analyzed.

## FastQC

FastQC (version 0.12.1) is a quality control tool that can detect potential issues with sequencing data such as poor sequence quality, overrepresentation of AT or GC base pairs, high N content, sequence length disribution, sequence duplicate levels (which is not a relevant metric for RNA-seq data because we expect certain genes be sequenced more often due to being more highly expressed) and the presence of adapter sequences (49). It has a graphical user interface but can also be used in the command line. This flexibility and the easy to read html report output make it a very useful tool for analyzing the quality of Next Generation Sequencing data (49). In this analysis it was used to assess potential problems with RNA-seq data, primarily to check the sequence quality and if the removal of N-rich sequences and adapter containing sequences by the company BGI was successful.

## Qualimap

Qualimap2 (version 2.3) takes a bam file from a splice aware aligner (in this case HISAT2) and a gtf file countaining the genome’s features (transcripts, their names and their genomic position) as input for RNA-seq analysis (50, 51) . Similarly to FastQC, it offers both a graphical user interface (GUI) and a command line interface, making it more accessible because it can accommodate both people preferring a GUI and people preferring to work in the command line. For this study, the RNA-seq mode was used, but the program can also analyze whole-genome, whole-exome and ChIP-seq data. As output, qualimap provides an html file that contains information about the alignment (number of mapped reads, not aligned reads etc), genomic origin of the reads (intron, exon, intergenic) and a junction analysis.

## FastQscreen

FastQscreen (version 0.15.3) is another quality control tool (52). In combination with an alignment software (Bowtie, Bowtie2 or BWA) and the genomes of other organisms, it can detect the presence of foreign nucleotide sequences and thus provide information if there is contamination in the sample provided and if there is, how badly the sample is contaminated (52). In this project, FastQscreen was used with Bowtie2 (53) indices built with the genomic data of *Homo sapiens (GRCh38), Escherichia coli (GCA001606525.ASM160652v1), Arabidopsis thaliana (TAIR10), Caenorhabditis elegans (WBcel235),* Saccharomyces *cerevisiae (R64-1-1), Candida albicans 19F (GCA_000775445), Aspergillus fumigatus (ASM265v1)* and *Aspergillus niger (ASM285v2)* taken from the Ensembl database (54)*.*

## Reference of detailed methodology

1. Intel Corp. 2024. Intel® Core^TM^ i9-13900K Processor. https://www.intel.com/content/www/us/en/products/sku/230496/intel-core-i913900k-processor-36m-cache-up-to-5-80-ghz/specifications.html. Retrieved 24 July 2024.

2. Malik M. 2024. HWinfo. Software Download. https://www.hwinfo.com.

3. Hannaford T. 2024. July 2024 Update on Instability Reports on Intel Core 13th and 14th Gen Desktop Processors. Intel Community Forum. https://community.intel.com/t5/Processors/July-2024-Update-on-Instability-Reports-on-Intel-Core-13th-and/m-p/1617113#M74792. Retrieved 24 July 2024.

4. Oracle. 2024. Oracle VM Virtual Box User Manual. Oracle.

5. Chambers J, Dalgaard P, Gentleman R, Hornik K, Ihaka R, Kalibera T, Lawrence M, Ligges U, Lumley T, Maechler M, Meyer S, Murrel P, Plummer M, Ripley B, Sarkar D, Temple Lang D, Tierney L, Urbanek S. 2024. R: A Language and Environment for Statistical Computing. R Foundation for Statistical Computing, Vienna, Austria.

6. Love MI, Huber W, Anders S. 2014. Moderated estimation of fold change and dispersion for RNA-seq data with DESeq2. Genome Biol 15:550.

7. Soneson C, Love MI, Robinson MD. 2015. Differential analyses for RNA-seq: transcript-level estimates improve gene-level inferences. F1000Res 4:1521.

8. Warnes G, Bolker B, Bonebakker L, Gentleman R, Huber W, Liaw A, Lumley T, Maechler M, Magnusson A, Moeller S, Schwartz M, Venables B. 2024. gplots. R 3.1.3.1.

9. Gu Z, Eils R, Schlesner M. 2016. Complex heatmaps reveal patterns and correlations in multidimensional genomic data. Bioinformatics 32:2847–2849.

10. Gu Z. 2022. Complex heatmap visualization. iMeta 1:e43.

11. Neuwirth E. 2022. RColorBrewer: ColorBrewer Palettes (1.1-3). R.

12. Stephens M. 2016. False discovery rates: a new deal. Biostat kxw041.

13. Van Rossum G, Drake FL. 2009. Python 3 Reference Manual. CreateSpace.

14. Cock PJA, Antao T, Chang JT, Chapman BA, Cox CJ, Dalke A, Friedberg I, Hamelryck T, Kauff F, Wilczynski B, De Hoon MJL. 2009. Biopython: freely available Python tools for computational molecular biology and bioinformatics. Bioinformatics 25:1422–1423.

15. Cole D. 2003. csv: Fast CSV Parser for Python. Python.

16. Rubinsteyn A. 2024. gtfparse (2.5.0). Python.

17. Hunter JD. 2007. Matplotlib: A 2D Graphics Environment. Comput Sci Eng 9:90–95.

18. Harris CR, Millman KJ, Van Der Walt SJ, Gommers R, Virtanen P, Cournapeau D, Wieser E, Taylor J, Berg S, Smith NJ, Kern R, Picus M, Hoyer S, Van Kerkwijk MH, Brett M, Haldane A, Del Río JF, Wiebe M, Peterson P, Gérard-Marchant P, Sheppard K, Reddy T, Weckesser W, Abbasi H, Gohlke C, Oliphant TE. 2020. Array programming with NumPy. Nature 585:357–362.

19. Roeschke M, Van den Bossche J, Augspurger T, Rebak J, Ayd W, Mendel JB, Garcia M, Hawkins S, Peterson T, McMaster A, Sheppard K, Lustig I, Gorelli ME, Shardach R, Hoefler P, arbyshire J, Li F, Wörtwein T, Li T, Manley L, Tamir N. 2024. pandas-dev/pandas: Pandas (v2.2.2).

20. Sovner EB, Mariotti M. pyranges: GenomicRanges for Python.

21. Virtanen P, Gommers R, Oliphant TE, Haberland M, Reddy T, Cournapeau D, Burovski E, Peterson P, Weckesser W, Bright J, Van Der Walt SJ, Brett M, Wilson J, Millman KJ, Mayorov N, Nelson ARJ, Jones E, Kern R, Larson E, Carey CJ, Polat İ, Feng Y, Moore EW, VanderPlas J, Laxalde D, Perktold J, Cimrman R, Henriksen I, Quintero EA, Harris CR, Archibald AM, Ribeiro AH, Pedregosa F, Van Mulbregt P, SciPy 1.0 Contributors, Vijaykumar A, Bardelli AP, Rothberg A, Hilboll A, Kloeckner A, Scopatz A, Lee A, Rokem A, Woods CN, Fulton C, Masson C, Häggström C, Fitzgerald C, Nicholson DA, Hagen DR, Pasechnik DV, Olivetti E, Martin E, Wieser E, Silva F, Lenders F, Wilhelm F, Young G, Price GA, Ingold G-L, Allen GE, Lee GR, Audren H, Probst I, Dietrich JP, Silterra J, Webber JT, Slavič J, Nothman J, Buchner J, Kulick J, Schönberger JL, De Miranda Cardoso JV, Reimer J, Harrington J, Rodríguez JLC, Nunez-Iglesias J, Kuczynski J, Tritz K, Thoma M, Newville M, Kümmerer M, Bolingbroke M, Tartre M, Pak M, Smith NJ, Nowaczyk N, Shebanov N, Pavlyk O, Brodtkorb PA, Lee P, McGibbon RT, Feldbauer R, Lewis S, Tygier S, Sievert S, Vigna S, Peterson S, More S, Pudlik T, Oshima T, Pingel TJ, Robitaille TP, Spura T, Jones TR, Cera T, Leslie T, Zito T, Krauss T, Upadhyay U, Halchenko YO, Vázquez-Baeza Y. 2020. SciPy 1.0: fundamental algorithms for scientific computing in Python. Nat Methods 17:261–272.

22. Waskom M. 2021. seaborn: statistical data visualization. JOSS 6:3021.

23. Pedregosa F, Varoquaux G, Gramfort A, Michel V, Thirion B, Grisel O, Blondel M, Müller A, Nothman J, Louppe G, Prettenhofer P, Weiss R, Dubourg V, Vanderplas J, Passos A, Cournapeau D, Brucher M, Perrot M, Duchesnay É. 2012. Scikit-learn: Machine Learning in Python (4) https://doi.org/10.48550/ARXIV.1201.0490.

24. Sheppard K, Fulton C, Perktold J. 2024. statsmodels v0.14.2 (v0.14.2).

25. Bekatas M, Bozarth A, Charles E, Collonval F, Cryan M, Darian A, Fauske VT, Granger B, Grout J, Krassowski M, Klein M, Pena-Castellanos G, Perez F, Presedo-Floyd I, Silvester S, Tuloup J. 2024. Jupyter-Lab.

26. Anaconda Software distribution. 2016. Computer software Anaconda.

27. GraphPad Support. Will you port Prism or InStat to Linux? Does Prism work with the Windows emulator Wine? https://www.graphpad.com/support/faq/will-you-port-prism-or-instat-to-linux-does-prism-work-with-the-windows-emulator-wine/. Retrieved 20 August 2024.

28. Kolmogorov M, Bickhart DM, Behsaz B, Gurevich A, Rayko M, Shin SB, Kuhn K, Yuan J, Polevikov E, Smith TPL, Pevzner PA. 2020. metaFlye: scalable long-read metagenome assembly using repeat graphs. Nat Methods 17:1103–1110.

29. Mikheenko A, Prjibelski A, Saveliev V, Antipov D, Gurevich A. 2018. Versatile genome assembly evaluation with QUAST-LG. Bioinformatics 34:i142–i150.

30. Kim D, Paggi JM, Park C, Bennett C, Salzberg SL. 2019. Graph-based genome alignment and genotyping with HISAT2 and HISAT-genotype. Nat Biotechnol 37:907–915.

31. Ryngajłło M, Boruta T, Bizukojć M. 2021. Complete genome sequence of lovastatin producer *Aspergillus terreus* ATCC 20542 and evaluation of genomic diversity among *A. terreus* strains. Appl Microbiol Biotechnol 105:1615–1627.

32. Danecek P, Bonfield JK, Liddle J, Marshall J, Ohan V, Pollard MO, Whitwham A, Keane T, McCarthy SA, Davies RM, Li H. 2021. Twelve years of SAMtools and BCFtools. GigaScience 10:giab008.

33. Patro R, Duggal G, Love MI, Irizarry RA, Kingsford C. 2017. Salmon provides fast and bias-aware quantification of transcript expression. Nat Methods 14:417–419.

34. Li H. 2018. Minimap2: pairwise alignment for nucleotide sequences. Bioinformatics 34:3094–3100.

35. Li H, Groot Koerkamp R. ksw2. https://github.com/lh3/ksw2. Retrieved 19 July 2024.

36. Pertea M, Pertea GM, Antonescu CM, Chang T-C, Mendell JT, Salzberg SL. 2015. StringTie enables improved reconstruction of a transcriptome from RNA-seq reads. Nat Biotechnol 33:290–295.

37. Pertea M, Kim D, Pertea GM, Leek JT, Salzberg SL. 2016. Transcript-level expression analysis of RNA-seq experiments with HISAT, StringTie and Ballgown. Nat Protoc 11:1650–1667.

38. Benjamini Y, Hochberg Y. 1995. Controlling the False Discovery Rate: A Practical and Powerful Approach to Multiple Testing. Journal of the Royal Statistical Society Series B: Statistical Methodology 57:289–300.

39. Benjamini Y, Yekutieli D. 2001. The control of the false discovery rate in multiple testing under dependency. Ann Statist 29.

40. Love MI, Anders S, Huber W. 2024. Analyzing RNA-seq data with DESeq2. https://www.bioconductor.org/packages/release/bioc/vignettes/DESeq2/inst/doc/DESeq2.html.

41. Hokken M, Coolen J, Steenbreker H, Zoll J, Baltussen T, Verweij P, Melchers W. 2023. The Transcriptome Response to Azole Compounds in *Aspergillus fumigatus* Shows Differential Gene Expression across Pathways Essential for Azole Resistance and Cell Survival. JoF 9:807.

42. Jones P, Binns D, Chang H-Y, Fraser M, Li W, McAnulla C, McWilliam H, Maslen J, Mitchell A, Nuka G, Pesseat S, Quinn AF, Sangrador-Vegas A, Scheremetjew M, Yong S-Y, Lopez R, Hunter S. 2014. InterProScan 5: genome-scale protein function classification. Bioinformatics 30:1236–1240.

43. Pertea G, Pertea M. 2020. GFF Utilities: GffRead and GffCompare. F1000Res 9:304.

44. Shen W, Le S, Li Y, Hu F. 2016. SeqKit: A Cross-Platform and Ultrafast Toolkit for FASTA/Q File Manipulation. PLoS ONE 11:e0163962.

45. Li H. 2021. New strategies to improve minimap2 alignment accuracy. Bioinformatics 37:4572–4574.

46. Smolka M, Paulin LF, Grochowski CM, Horner DW, Mahmoud M, Behera S, Kalef-Ezra E, Gandhi M, Hong K, Pehlivan D, Scholz SW, Carvalho CMB, Proukakis C, Sedlazeck FJ. 2024. Publisher Correction: Detection of mosaic and population-level structural variants with Sniffles2. Nat Biotechnol https://doi.org/10.1038/s41587-024-02141-2.

47. Smolka M, Paulin LF, Sedlazeck FJ. 2023. Sniffles2 supporting matetial: VCF https://doi.org/10.5281/ZENODO.8144524.

48. Quinlan AR, Hall IM. 2010. BEDTools: a flexible suite of utilities for comparing genomic features. Bioinformatics 26:841–842.

49. Andrews S. 2010. FastQC: A Quality Control Tool for High Throughput Sequence Data [Online].

50. García-Alcalde F, Okonechnikov K, Carbonell J, Cruz LM, Götz S, Tarazona S, Dopazo J, Meyer TF, Conesa A. 2012. Qualimap: evaluating next-generation sequencing alignment data. Bioinformatics 28:2678–2679.

51. Okonechnikov K, Conesa A, García-Alcalde F. 2016. Qualimap 2: advanced multi-sample quality control for high-throughput sequencing data. Bioinformatics 32:292–294.

52. Wingett SW, Andrews S. 2018. FastQ Screen: A tool for multi-genome mapping and quality control. F1000Res 7:1338.

53. Langmead B, Salzberg SL. 2012. Fast gapped-read alignment with Bowtie 2. Nat Methods 9:357–359.

54. Martin FJ, Amode MR, Aneja A, Austine-Orimoloye O, Azov AG, Barnes I, Becker A, Bennett R, Berry A, Bhai J, Bhurji SK, Bignell A, Boddu S, Branco Lins PR, Brooks L, Ramaraju SB, Charkhchi M, Cockburn A, Da Rin Fiorretto L, Davidson C, Dodiya K, Donaldson S, El Houdaigui B, El Naboulsi T, Fatima R, Giron CG, Genez T, Ghattaoraya GS, Martinez JG, Guijarro C, Hardy M, Hollis Z, Hourlier T, Hunt T, Kay M, Kaykala V, Le T, Lemos D, Marques-Coelho D, Marugán JC, Merino GA, Mirabueno LP, Mushtaq A, Hossain SN, Ogeh DN, Sakthivel MP, Parker A, Perry M, Piližota I, Prosovetskaia I, Pérez-Silva JG, Salam AIA, Saraiva-Agostinho N, Schuilenburg H, Sheppard D, Sinha S, Sipos B, Stark W, Steed E, Sukumaran R, Sumathipala D, Suner M-M, Surapaneni L, Sutinen K, Szpak M, Tricomi FF, Urbina-Gómez D, Veidenberg A, Walsh TA, Walts B, Wass E, Willhoft N, Allen J, Alvarez-Jarreta J, Chakiachvili M, Flint B, Giorgetti S, Haggerty L, Ilsley GR, Loveland JE, Moore B, Mudge JM, Tate J, Thybert D, Trevanion SJ, Winterbottom A, Frankish A, Hunt SE, Ruffier M, Cunningham F, Dyer S, Finn RD, Howe KL, Harrison PW, Yates AD, Flicek P. 2023. Ensembl 2023. Nucleic Acids Research 51:D933–D941.
